# Nanoformulation of Spirooxindole and Methods for Treating Hepatocellular Carcinoma

**DOI:** 10.3390/pharmaceutics17010093

**Published:** 2025-01-12

**Authors:** Assem Barakat, Fardous F. El-Senduny, Mohammad Shahidul Islam, Abdullah Mohammed Al-Majid, Yaseen A. M. M. Elshaier, Eman A. Mazyed, Farid A. Badria

**Affiliations:** 1 Department of Chemistry, College of Science, King Saud University, P.O. Box 2455, Riyadh 11451, Saudi Arabia; mislam@ksu.edu.sa (M.S.I.); amajid@ksu.edu.sa (A.M.A.-M.); 2 Department of Pathology & Laboratory Medicine, Sylvester Comprehensive Cancer Center, Miller School of Medicine, Miami, FL 33136, USA; fxe123@med.miami.edu; 3 Department of Chemistry, Faculty of Science, Mansoura University, Mansoura 35516, Egypt; 4 Department of Organic and Medicinal Chemistry, Faculty of Pharmacy, University of Sadat City, Menoufiya 32958, Egypt; yaseen.elshaier@fop.usc.edu.eg; 5 Department of Pharmaceutical Technology, Faculty of Pharmacy, Kaferelsheikh University, Kaferelsheikh 33516, Egypt; eman_mazyad@pharm.kfs.edu.eg; 6 Department of Pharmacognosy, Faculty of Pharmacy, Mansoura University, Mansoura 35516, Egypt

**Keywords:** spirooxindole, nanoformulation, p53-MDM2, cancer research, hepatocellular carcinoma

## Abstract

**Objectives:** This in vivo study introduces a newly developed spirooxindole derivative that is deemed safe and effective as a potential targeted therapy for various cancers. **Methods:** Extensive in vivo investigations, including histopathology, immunohistochemistry, and molecular biology, validated its potential for further preclinical and clinical exploration, necessitating comprehensive examinations of its bioavailability, pharmacodynamics, and pharmacokinetics. Additionally, this study involves the development of a commercially viable proniosomal drug delivery system for the compound, facilitating controlled drug release. **Results:** The data revealed efficacy of spirooxindole derivative in halting the progression of liver cancer, metastasis, and portal vein thrombosis, with potential implications for enhancing regeneration and recovery of early-stage cancer cells in multiple organs, thereby improving recovery rates and remission among cancer patients. The proniosomes, loaded with the compound, exhibited high entrapment efficiency and prolonged drug release rates of up to 12 h in vitro. The optimized formula demonstrated superior drug release percentages and stability compared to conventional niosomes. Further analysis via FTIR and DSC confirmed the absence of chemical interactions and proper entrapment of the compound within the nanovesicles, indicating a stable and effective drug delivery system. **Conclusions:** This study presents a novel, safe, and effective chemical entity of spirooxindole derivatives for further preclinical and clinical studies.

## 1. Introduction

P53 plays a crucial and indispensable role in regulating various interconnected biological processes, including glycolysis, the cell cycle, apoptosis, autophagy, and cell differentiation. Its activity is finely modulated at multiple levels, including transcription, translation, and stability [1]. Its protein stability is regulated by human homologue of mouse double minute 2 (MDM2). MDM2 is an E3 ubiquitin ligase whose expression is regulated by p53 through an autoregulatory feedback mechanism. It promotes p53 degradation by tagging it with ubiquitin for proteasomal breakdown. Additionally, MDM2 binds to the N-terminal transactivation domain of p53, preventing its interaction with target gene sequences and suppressing its transcriptional activity. This regulatory loop ensures controlled p53 activity under normal cellular conditions [2,3,4]. Mutated or deleted p53 protein is linked to 50% of cancer types in human [5,6]. The activity of wild type p53 in cancer is inhibited by the overexpression of MDM2. Activation of p53 through the blocking of MDM2-p53 interaction is an interesting and attracting target for scientists [7,8,9,10,11,12,13,14,15]. Several discovered inhibitors are in clinical trials [16]. However, their toxicity on platelets limited their clinical application. Therefore, there is still need to discover more selective and effective drugs [17,18,19,20].

Spirooxindoles possess a distinctive and appealing scaffold that effectively fits into the binding domain of MDM2, thereby obstructing their interaction. This blockade subsequently triggers the activation and translocation of p53 to the nucleus [12,13]. The nuclear translocation of p53 triggers the activation of its target genes, which play pivotal roles in regulating various signaling pathways.

**Scheme 1:**
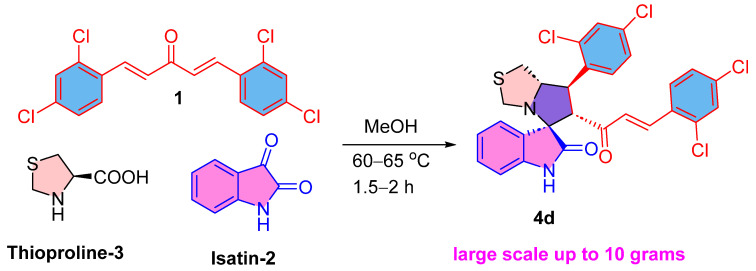
The synthesis of drug candidate **4d**.

In vitro study of the anticancer activity of compound **4d** (Scheme 1) revealed that it has a broad anticancer activity against colon, liver, and prostate cancer with a selectivity index greater than two [7]. Additionally, compound **4d** led to the activation of p53 and overexpression of cell cycle inhibitor p21 gene in the treated colon cancer cells, inhibited colony formation, and would healing. Moreover, compound **4d** showed induction of apoptosis through the inhibition of antiapoptotic Bcl-2 and activation of Bax. Based on the interesting results, this in vivo study was designed to investigate the anticancer activity of compound **4d** against CCl_4_-induced liver cancer.

Physico-chemical properties of the target compound strongly predict the pharmacokinetics of the novel drug candidate, which is very great challenge in the drug development process. Lipophilicity is among the fundamental physicochemical characteristics of a novel chemical entity. Within biological systems, it exerts a significant influence on the pharmacokinetics and pharmacodynamics of potential drug candidates [2,3,4]. Hence, assessing lipophilicity in the early stages of development can substantially mitigate issues related to poor ADME (absorption, distribution, metabolism, and excretion) properties of new drug candidates and/or enhance their efficacy. This underscores the paramount importance of this parameter. Lipophilicity of a compound is typically quantified as the logarithm of its partition coefficient [5,6,7].

The Rapid Overlay of Chemical Structures (ROCS) tool is employed to assess the similarity between molecules by analyzing their three-dimensional shapes [8]. This method of shape similarity comparison finds various applications, notably in lead-hopping and structural predictions.

The method implemented in this work is to explain the compounds activity based on their 3D structures according to their common pharmacophoric features as a lead-hopping application [9,10]. Shape demonstrates favorable neighborhood behaviors, indicating that high similarity in shape often correlates with high similarity in biology, rather than merely reflecting similarities in 2D structures [11].

However, poor solubility of compound **4d** could result in poor bioavailability, thus hindering its clinical applications. Thus, using nanocarriers are highly warranted to overcome these challenges. Nanocarriers offer a means to enhance the therapeutic effectiveness of drugs while minimizing their adverse effects through controlled drug release. Liposomes, which are phospholipid-based nanovesicles, have the capability to encapsulate both hydrophilic and lipophilic drugs. However, they have limited chemical and physical stability [21]. Niosomes are alternate systems that are used to overcome the limitations of liposomes. Niosomes are nanovesicles prepared using non-ionic surfactants that are more stable and lower in cost when compared to the liposomes. Nevertheless, niosomes have some physical stability problems in aqueous dispersions, such as vesicular aggregation, fusion, and leakage [22].

Proniosomes are formulated either as dry, free-flowing powders, where non-ionic surfactants are coated onto a water-soluble carrier or as liquid crystals of surfactants with a gel-like structure. Both forms of proniosomes can be easily converted into niosome suspensions before use by reconstituting them with aqueous media. Proniosomes have the potential of addressing the physical instability issues associated with niosomes and provide greater convenience in terms of transportation and storage [23]. In this study, we performed extensive in vivo investigations of spirooxindole analog **4d**, including histopathological, immunohistochemical, and molecular biology analyses. Our results support its potential for further preclinical and clinical development, emphasizing the importance of in-depth assessments of its bioavailability, pharmacodynamics, and pharmacokinetics.

## 2. Materials and Methods

### 2.1. Chemistry

The drug candidate was re-synthesized in significant quantities for animal studies and nanformulation in accordance with our previously patented methodology [7,8] (Scheme 1); the chemical structure was reconducted NMR analysis, confirming that the results matched the reported data [7,8].

### 2.2. Animal Design

In vivo study was carried out in Male Balb/c mice weighing (15–25 g). All mice were purchased from Medical Experimental Research Center (MERC), Mansoura University, Mansoura, Dakahlia Governorate, Egypt. Mice were acclimatized for one week in the new animal care facility, Zoology Department, Mansoura University, Egypt for one week under specific pathogen-free conditions (SPF) and provided with conventional food and drinking water before starting the study. All animal experiments were carried out following the guidelines of the faculty of Science, Mansoura University, Mansoura, Dakahlia Governorate, Egypt.

### 2.3. Evaluation of the Lethal Dose (LD50)

The lethal dose was evaluated according to Dose-Probing Tests [24,25,26]. Mice (3 per group) were divided into groups in order to evaluate LD50. Each group received either different concentration of compound **4d** starting from 10 ng/Kg to 200 mg/Kg/corn oil or 10%DMSO/corn oil by IP injection [27]. The weight and physical symptoms were monitored for 14 days. After that, all groups were sacrificed, and liver, kidney, heart, and spleen were collected for histological evaluation of the toxicity of compound **4d**. Blood was collected for evaluation of the effect of compound **4d** on the liver and kidney function.

### 2.4. Establishment of the Liver Dysplasia (Premalignant) and Treatment [28]

Mice (25) were treated with CCl_4_ (LOBA Chemie Pvt Ltd., Mumbai, India, 1:5 in corn oil/twice a week) via intraperitoneal injection for 24 weeks. After that, mice were distributed into 5 groups (5 per each); gp1, treated with the solvent 10% DMSO [27,29]; gp2, treated with low dose of compound **4d** (50 mg/kg/daily) for 14 days; gp3, treated with high dose of compound **4d** (200 mg/kg) for 6 h; gp4, treated with high dose of compound **4d** (200 mg/kg) for 24 h; gp5, treated with high dose of compound **4d** (200 mg/kg) for 48 h. At the end of each treatment, mice were sacrificed, and blood and liver were collected. Liver samples were washed in ice cold 1x PBS and fixed in 10% buffered formalin until examination.

### 2.5. Biochemical Markers

Blood was centrifuged to collect either serum or plasma for downstream biochemical analysis. Blood chemistry was performed to detect the level of serum alanine aminotransferase (ALT), serum aspartate aminotransferase (liver function test), and serum creatinine (kidney function test). Complete blood count analysis was carried out to detect the effect of compound **4d** treatment on platelets count because it is reported in the literature that p53-MDM2 inhibitors cause high grade thrombocytopenia (low number of platelets) [29].

### 2.6. Histopathology and Immunohistochemistry

Formalin-fixed liver specimens were embedded in paraffin, and sections were prepared for hematoxylin and eosin (H&E) staining. The slides were examined for hepatic architecture, degenerative changes, inflammatory changes, hepatocytes necrosis, mitosis, dysplastic changes, and malignant transformation. Four-micrometer-thick sections were prepared on coated slides. The sections were de-paraffinized in xylene then rehydrated. Endogenous tissue peroxidase was blocked by incubating the slides for 5 min in 0.3% hydrogen peroxide. Antigen retrieval was performed by heating in citrate buffer (pH 6.0) in a microwave. Then, sections were incubated with monoclonal mouse p53 (clone DO-7, Dako, Santa Clara, CA, USA), rabbit polyclonal caspase 3 antibody (CPP32) Ab-4 (Thermo Scientific, Waltham, MA, USA) or with monoclonal mouse Ki-67 antigen (clone MIB-1, Dako, Santa Clara, CA, USA). Slides were counterstained with hematoxylin and then dehydrated in alcohol and cleared xylene. The immunohistochemical and pathological changes were examined by light microscopy (binocular, Olympus, Hamburg, Germany). Images were taken using a digital camera (Canon 5 mega pixels, 3.2× optical zoom (Canon, Tokyo, Japan)). A total of 10 fields per section were randomly selected and examined.

### 2.7. Statistical Analysis

Statistical analysis was conducted using SPSS 19 software for Microsoft Windows (SPSS Inc., Chicago, IL, USA). The quantitative analysis of the number of hepatocytes with positive nuclear Ki-67 staining was assessed using a one-way analysis of variance (ANOVA) with Duncan’s multiple comparisons of the means to compare differences between means. Data were presented as means ± standard errors, and differences between means were considered significant when *p* < 0.05. Blood chemistry and CBC data were expressed as means ± standard deviation (SD), and differences between multiple groups were compared using *t*-tests. Results with a *p* value less than 0.05 were deemed statistically significant.

### 2.8. ROCS Study

ROCS was computed using the ROCs application from OpenEye scientific software (OpenEye-application-2024; 30-7-2024. The Laboratory of Yaseem Elsahier, University of Sadat City, Egypt) [30]. Compound **4d** was designated as the query molecule, while a compound library of spirooxindoles served as the database file. vROCS was utilized to execute and assess/visualize the outcomes. The ROCS application scanned the database using the query to identify molecules with similar shapes and colors. Compound conformers were evaluated based on the Gaussian overlap with the query, and the optimal scoring parameter was determined to be the Tanimoto Combo score (shape + color), with the highest score indicating the best match with the query compound.

### 2.9. Formulation of ***4d***-Loaded Proniosomes

#### 2.9.1. Preliminary Screening Study

A Preliminary screening study was conducted to choose the most appropriate formulation parameters. The dry proniosomes are hydrated before administration to be converted to the niosomal dispersion. Hence, short hydration time and hydration at room temperature would increase the applicability of the formulation and patient compliance. In order to choose the proper surfactant and the appropriate hydration time, the proniosomal formulations have been prepared using 5 mg/mL of compound **4d**, maltodextrin as the carrier and 140:60 µmolar ratio of surfactant to cholesterol (CHOL).

The first step of the prescreening test was choosing the surfactant that could formulate proniosomes that are rehydrated at 25 °C. Two types of surfactants Sorbitan monooleate (Span 80) and Sorbitan monostearate (Span 60) were employed for the preparation of **4d**-loaded proniosomes. The proniosomal formulations were tested for vesicle formation at 25 °C using optical microscope (Coslabs micro, Ambala Cantt, India) with magnification of (40×).

The next step was studying the proper time of hydration of the proniosomal powder. Three different hydration times (2, 5, and 30 min) were investigated for hydration of the selected proniosomes by measurement of EE%.

#### 2.9.2. Fabrication of **4d**-Loaded Proniosomes Using the Slurry Method

Proniosomes of **4d** compound were formulated by the slurry method. Accurately weighed amounts of **4d** (5 mg/mL), non-ionic surfactant and CHOL were dissolved in 10 mL chloroform. The slurry is formed by introducing the resultant solution into a round-bottom flask containing the hydrophilic carrier. The organic solvent was removed at 60 ± 2 °C under reduced pressure (16 mm Hg) using a rotary evaporator (Buchi rotavapor R-3000, Flawil, Switzerland) that was adjusted to 80 rpm until the mass in the round-bottom flask becomes a completely dry free-flowing product. The formulated proniosomes were stored in a desiccator to be used for further studies [31].

### 2.10. Experimental Design of Proniosomes of ***4d*** Using 2^3^ Factorial Design

Proniosomes of **4d** were fabricated according to a two-level (2^3^) factorial design using Design-Expert software, Version 7 (Stat-Ease Inc., Minneapolis, MN, USA) to explore the impact of different formulation variables on the characteristics of different proniosomal vesicles. In this design, the type of carrier (X1), the ratio of Sorbitan monooleate to CHOL (X2) and the volume of hydration (X3) were selected as the independent factors. Each variable was screened at three levels: the lower, the middle, and the upper levels (−1, 0, and +1), respectively. The percentage of drug released after 12 h (Q_12h_, Y1) and entrapment efficiency (EE%, Y2) were selected as the responses [32].

Goodness of fit of the model to the experimental data was tested by determination of the coefficient of determination (R^2^), adjusted R^2^, and predicted R^2^ that are considered statistical measures of closeness of the data to the fitted regression line. Moreover, the analysis of variance (ANOVA) was used for statistical analysis and determining the significance of the obtained results on the basis of F statistics and the *p*-value. The *p*-value is used to check the significance of each coefficient; a *p*-value that is lower than 0.05 is considered to be significant at a level of significance α = 0.05, showing that the corresponding factor is significant, and the null hypothesis could be rejected. The smaller the *p*-value, the more significant is the tested coefficient [33].

### 2.11. Preparation of Proniosome-Derived Niosomal Dispersions of ***4d***

The niosomal dispersion of **4d** was prepared by hydration of the proniosomal powder using distilled water at 25 °C ± 2 °C for 2 min using a vortex mixer (BOECO, Hamburg, Germany). The niosomal dispersion was then sonicated using a bath sonicator (Elmasonic E 30 H, Elma, Singen, Germany) for 5 min and left in a refrigerator at 4 °C to mature overnight to be used for further studies [34].

### 2.12. Determination of Drug Content, Drug-Loading Capacity, and Entrapment Efficiency of ***4d***-Loaded Proniosomes

EE% of proniosome-derived niosomal dispersions of **4d** was determined using the indirect method. The ultracentrifugation method was used for separation of the free (un-entrapped) drug using a cooling ultracentrifuge (Biofuge, primo Heraeus, Hanau, Germany) at 14,000 rpm for 1 h at 4 °C [35]. The supernatant was separated and analyzed for drug content. The encapsulation efficiency and drug-loading capacity (DLC) of **4d**-loaded proniosomes were calculated as follows:

EE (%) = (A1 − A2) × 100/A1

where A1  =  Initial amount of drug, A2  =  Amount of drug in the supernatant

% DLC = (The encapsulated **4d** amount) × 100/(The weight of proniosomes)

For the determination of total drug content (unentrapped + entrapped), 1 mL of proniosome-derived niosomal dispersion was disrupted by isopropyl alcohol (100 mL) [36]. The samples were filtered through a 0.45 µM membrane filter and analyzed for drug content using T80+ UV/VIS PC Spectrophotometer (PG Instruments Ltd., Woodway Lane, Wibtoft, UK).

### 2.13. In Vitro Release of ***4d***-Loaded Proniosomes

In vitro release of proniosome-derived niosomal dispersions of **4d** was studied using modified Franz diffusion cells. The cellulose membrane was hydrated using a phosphate-buffered solution (pH = 7.4) for 24 h at room temperature. The prehydrated cellulose membrane was mounted precisely between the donor and receptor compartments. The receptor medium was 20 mL phosphate-buffer solution (pH 7.4) containing 1% sodium lauryl sulfate (SLS) to maintain sink conditions. The receptor compartment was kept at 37 ± 0.5 °C and stirred at 100 rpm using a magnetic stirrer. An accurate volume (1 mL) of the proniosome-derived niosomal dispersions containing entrapped **4d** was placed over the cellulose membrane in the donor compartment. 200 µL sample was taken at predetermined time intervals, and the receiver cell was replenished with an equal volume of fresh phosphate-buffer solution. The withdrawn samples were analyzed for drug content. Triplicate measurements were conducted for each study, and the data were expressed as the mean values ± SD [37].

The mechanism of the in vitro release of **4d**-loaded proniosomes was explored by studying the release kinetics through treating the data of drug release using different mathematical models, and the model having the highest coefficient of determination (R^2^) was selected to describe the in vitro release profile [38].

### 2.14. Statistical Optimization of ***4d***-Loaded Proniosomes

Selection of the optimized **4d**-loaded proniosomal formula depends on determination of the desirability index that describes the desirable range for each response. The value of desirability index lies between 0 and 1. The value 0 corresponds to an undesirable response, while the value 1 expresses an optimal performance of the studied factors [33]. In the present model, the choice of the optimized proniosomal formula depends on maximizing both Q_12h_ and EE%. The optimized **4d**-loaded proniosomal formula was evaluated by further characterization tests.

### 2.15. Comparative Study of the Optimized Proniosomal Formula and the Conventional Niosomes

A comparative study was performed between the optimized proniosomal formula and the corresponding niosomal dispersion through evaluation of EE%, Q_12h_, and stability study.

#### 2.15.1. Formulation of **4d**-Loaded Niosomes

**4d**-loaded niosomes were formulated using thin film hydration method [39]. Briefly, the drug (5 mg/mL), Sorbitan monooleate, and CHOL were mixed at the selected molar concentrations and dissolved in 10 mL chloroform in a round-bottom flask. The organic solvent was then removed under vacuum in a rotary evaporator at 60 ± 2 °C under reduced pressure (16 mm Hg) using a rotary evaporator (Buchi rotavapor R-3000, St. Gallen, Switzerland), leaving a thin lipid film on the walls of the flask. The thin lipid film was rehydrated by 10 mL phosphate buffer (pH 7.4) and rotated for 30 min at 60 ± 2 °C. The resultant niosomal dispersion was left to mature overnight and stored in a refrigerator at 4 °C for further characterization.

#### 2.15.2. Evaluation of EE% and In Vitro Release of **4d**-Loaded Niosomes

EE% and the in vitro release profile of **4d**-loaded niosomes were studied as previously described. The in vitro release profile of the **4d**-loaded niosomes was compared to that of the optimized proniosomal formula according to the similarity factor test in which the in vitro release profiles are declared similar if the value of f2 is between 50 and 100. f2 is calculated according to the following equation [40]:

$$F2=50.log\left\{{\left[1+\frac{1}{n}\;\sum_{t=1}^{n}{\left(Rt-Tt\right)}^{2}\right]}^{-0.5}\right\}100$$

where *Rt* and *Tt* are the % drug released at time t from the optimized **4d**-loaded proniosomal formula and the corresponding niosomes, respectively, and *n* is the number of sampling points.

#### 2.15.3. Stability Study of the Optimized **4d**-Loaded Proniosomal Formula and the Corresponding Niosomes

Stability testing is a critical parameter that describes the effect of storage on the stability and characteristics of the formulation. Both the **4d**-loaded proniosomal formula and the corresponding niosomes were kept in tightly closed containers and stored in a refrigerator at 4 °C for a period of 3 months. The residual drug content and EE% of the stored formulations were evaluated at predefined time intervals as a measure of the stability of the nanovesicles [41,42].

### 2.16. Evaluation of the Selected Formula of ***4d***-Loaded Proniosomes

The optimized formulation of **4d**-loaded proniosomes was characterized through assessment of micromeritic properties, Fourier-transform infrared spectroscopy (FTIR), differential scanning calorimetry (DSC), morphological examination, and determination of vesicle size and zeta potential.

#### 2.16.1. Micromeritic Properties

The flow properties of the selected proniosomal powder were assessed by measuring the angle of repose using the funnel method for both the carrier and the optimized proniosomal powder. In brief, the carrier or proniosome powder was poured through a funnel positioned at a specific height, ensuring that the outlet orifice was 5 cm above a surface covered with graph paper. As the powder flowed from the funnel, it accumulated on the paper surface, forming a cone shape [43]. The angle of repose was subsequently calculated by measuring the height of the powder pile (h) and the radius (r) of its base. The angle of repose was determined using the following formula:

Tan θ = h/r

(θ is the angle of repose, h is the height of the powder pile, and r is the radius of the base of powder pile).

#### 2.16.2. Scanning Electron Microscopy (SEM)

The morphological characteristics of the reconstituted proniosomal formula were analyzed using a scanning electron microscope (SEM) (Jeol, JSM-6360, Tokyo, Japan). The diluted dispersion was applied onto an aluminum stub using double-sided adhesive carbon tape and dried under reduced pressure. Following this, the sample was coated with a layer of gold film. The coated specimen was then examined and photographed using SEM [44].

#### 2.16.3. Vesicle Size and Zeta Potential Determination

The vesicle size and zeta potential were assessed to investigate the colloidal properties of the reconstituted proniosomal formula. The dynamic light scattering (DLS) was used to analyze the size distribution of the vesicles. DLS is based on the scattering of light by vesicles in the reconstituted proniosomal dispersion in different directions. The rate of fluctuation in the intensity of scattered light is related to the Brownian motion of the vesicles, which is used to calculate their vesicle size.

The reconstituted proniosomal formula was appropriately diluted with distilled water. Subsequently, a NICOMP 380 ZLS zeta potential/particle sizer (PSS Nicomp, Santa Barbara, CA, USA) was employed to determine the vesicle size and zeta potential. The average of three measurements was recorded [45].

#### 2.16.4. Fourier Transform Infrared Spectroscopy (FTIR)

The possible interactions between the **4d** and different additives were studied by FT-IR using the FT-IR spectrometer (FTIR Shimadzu 8300, Kyoto, Japan). Samples of **4d**, different excipients, plain proniosomes and the optimized **4d**-loaded proniosomal formula were used. All these samples were grounded and mixed with potassium bromide to form pellets in a hydraulic press (Kimaya Engineers, Maharastra, India). The spectral range of the pellets was 4000–400 cm^−1^ [46].

#### 2.16.5. Differential Scanning Calorimetry (DSC) Study

DSC was used to study the change in the crystalline state of **4d** in the optimized proniosomal formula using a Shimadzu DSC 60 (Kyoto, Japan). The samples of **4d**, plain proniosomes, and the chosen **4d** proniosomal formula were heated in aluminum pans in the range of 20–260 °C with a heating rate of 10 °C/ min, and the DSC thermograms were recorded [47].

## 3. Results

### 3.1. Evaluation of Compound ***4d*** Toxicity in Mouse Model

**Figure 1:**
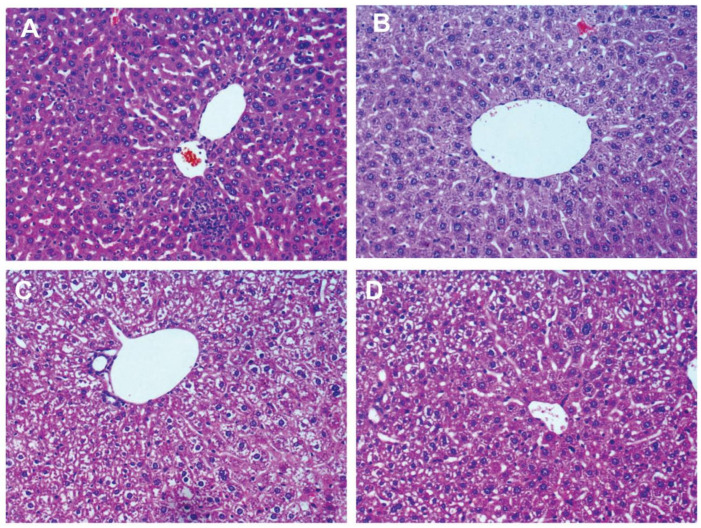
H&E staining of normal liver showed normal liver architecture with normal liver cells and lobules in all tested groups: (**A**) 10% DMSO, (**B**) 50 mg/kg compound **4d**, (**C**,**D**) 200 mg/kg compound **4d**. Compound **4d** did not cause any morphological changes in hepatic cells (×200).

In order to evaluate the tolerated dose of compound **4d**, mice were intraperitoneally injected with different doses and the physical symptoms were evaluated daily for 14 days. The maximum dose that used was 200 mg/kg because of the solubility limitation in DMSO. DMSO was adjusted to the tolerated dose 10% in corn oil [27,29]. Compound **4d** did not cause any change in the behavior, feeding, or drinking of the mice. Moreover, the complete blood count showed that there is not any aberrant or significant change in the platelets count (Supplementary Materials Table S1). Additionally, the effect of compound **4d** treatment on liver and kidney function was evaluated by measuring the level of aspartate aminotransferase (AST), alanine aminotransferase (ALT), and serum creatinine, respectively. The analysis revealed that AST, ALT, and creatinine levels were not changed by compound **4d** treatment (Supplementary Materials Table S1). This finding of the safety of compound **4d** on healthy mice was confirmed by histopathology analysis for fixed liver, heart, kidney, and spleen specimens to examine hepatic architecture, degenerative changes, inflammatory changes, hepatocytes necrosis, mitosis, dysplastic changes, and malignant transformation. Hematoxylin and eosin staining showed normal liver architecture without any degenerative changes after compound **4d** treatment for 14 days (Figure 1). Moreover, compound **4d** did not cause any pathological alteration in kidney, heart, or spleen (Supplementary Materials Figures S1–S3). These data confirm the applicability and safety of compound **4d**.

### 3.2. Evaluation of the Liver Histopathology After Compound ***4d*** Treatment

**Figure 2:**
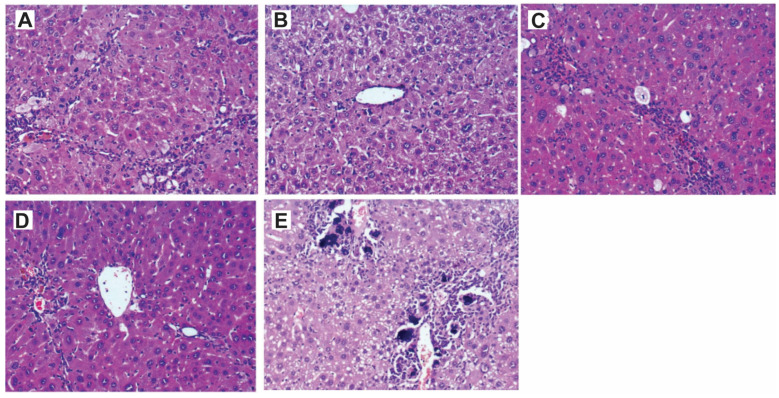
H&E staining of liver tissues showing the efficacy of compound **4d** in ameliorating the liver damage caused by CCl_4_ treatment. (**A**) Slides for CCl_4_-treated mice showed large cell dysplasia (premalignant). The liver architecture was disturbed, and there were cirrhotic nodules with bridging fibrosis. (**B**) Slides showed preserved liver architecture of the group treated with 50 mg/kg/daily for 14 days with mild inflammatory cellular infiltrate in portal tracts, and the liver cells showed no nuclear changes and no mitotic activity. (**C**) The group treated with one dose of 200 mg/kg for 6 h, the same histopathological pattern of (**A**) with anisonucleosis (nuclei vary in size) and minimal mitotic activity. (**D**) The group treated with a single dose of 200 mg/kg for 24 h showed mild inflammatory cellular infiltrate in portal tracts and the nuclei showed minimal anisonucleosis and lower mitotic activity. (**E**) The group treated with a single dose of 200 mg/kg for 48 h showed cirrhotic nodules with bridging fibrosis and intense inflammatory cellular infiltrate in portal tracts. The liver cells showed microvascular steatosis with numerous calcification foci were present in the portal tracts. The mitotic activity was low (HE, ×: 100).

An in vivo model was carried out to test the efficacy of compound **4d** in treatment of liver cancer by applying two regimens of compound **4d**: a single dose of 200 mg/kg and the treated mice were sacrificed after 6, 24, or 48 h or a daily dose of 50 mg/kg for 14 days. The liver cancer was induced chemically by CCl_4_ (1:5 dilution in corn oil, twice per week). H&E slides for CCl_4_-treated mice showed large cell dysplasia (premalignant) [28]. The liver architecture was disturbed, and there were cirrhotic nodules with bridging fibrosis (Figure 2A). The liver cells showed piecemeal necrosis (interface hepatitis). The liver cells also showed vacuolar degeneration with enlarged cytoplasm and nuclei with variability in size. The mitotic activity was high 12 mitotic figure/10 high power field (H.P.F.) with a high level of inflammatory infiltration in both portal tracts and parenchyma due to the accumulation of leukocytes (Figure 2A). The H&E slides for the daily dose of 50 mg/kg compound **4d**-treated group showed multiple liver lobes with confluent liver cell necrosis and excess inflammatory cellular infiltrate. However, other areas showed preserved liver architecture with mild inflammatory cellular infiltrate in portal tracts. Moreover, the liver cells showed no nuclear changes and no mitotic activity (Figure 2B), indicating the ability of compound **4d** to treat or stop the progression of hepatic cancer. This improvement in liver architecture was significantly reflected on the serum level of liver enzymes AST (*p* ≤ 0.001) and ALT (*p* ≤ 0.001) in comparison to DMSO-treated mice (Supplementary Materials Table S2).

To our knowledge, the application of a single-dose regimen has been used by two scientific research groups [9,48]; so, here in this study, compound **4d** efficacy was also tested at a single dose of 200 mg/kg for different time points (6, 24 or 48 h). After 6 h, the same histopathological pattern of the liver prepared from mice treated with CCl_4_ was observed where photomicrograph showed bridging fibrosis, high inflammatory infiltrate, and anisonucleosis (nuclei varies in size) with minimal mitotic activity (1/10 H.P.F.), indicating that this single high dose of compound **4d** inhibited mitosis (Figure 2C). On the other hand, longer incubation with the single dose showed a different pattern of liver architecture where the sections revealed some cirrhotic nodules, but there was mild inflammatory cellular infiltrate in portal tracts, and the nuclei showed minimal anisonucleosis and lower mitotic activity (1/10 H.P.F.) (Figure 2D). The liver of mice treated for two days showed cirrhotic nodules with bridging fibrosis and intense inflammatory cellular infiltrate in portal tracts. The liver cells showed microvascular steatosis with numerous calcification foci in the portal tracts. The mitotic activity was low (2/10 H.P.F.) (Figure 2E). The finding results from the single dose confirm the ability and efficacy of compound **4d** in treating the liver, and the histopathological results were in accordance with significantly lower levels of serum ALT (*p* ≤ 0.001) and AST (*p* ≤ 0.001) of the treated groups (Supplementary Materials Table S2).

### 3.3. Activation of p53 by Compound ***4d*** Was Observed in In Vivo Study

**Figure 3:**
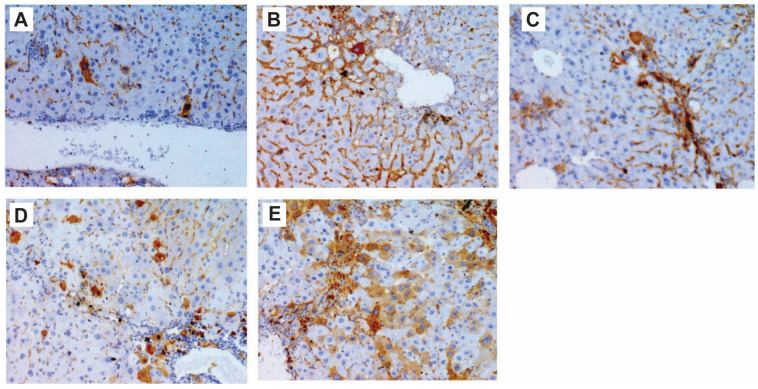
Immunohistochemistry showing activation of p53 after compound **4d** treatment in CCl_4_-treated mice. (**A**) Group treated with CCl_4_ showed low level of p53, while (**B**) group treated daily with low dose of compound **4d** for 14 days showed higher level of detected p53 in liver sections. (**C**) Group treated with one dose of 200 mg/kg for 6 h. (**D**) Group treated with one dose of 200 mg/kg for 24 h. (**E**) Group treated with one dose of 200 mg/kg for 48 h. (**C**–**E**) groups showed activation of p53 in a time-dependent manner (200×).

In order to evaluate the effect of compound **4d** on the activation of p53 in animal model, mice were treated as mentioned in the Material and Methods then immunohistochemistry was performed to detect the level of p53 in the fixed liver sections. It was observed that compound **4d** treatment at the two tested regimens, either low or high dose, was able to activate p53 (Figure 3). These results confirmed the efficacy of compound **4d** in treating the liver through the inhibition of MDM2-p53 interaction, which is in accordance with the in vitro study by flow cytometric analysis and molecular docking [7].

### 3.4. Compound ***4d*** Induced Apoptosis

**Figure 4:**
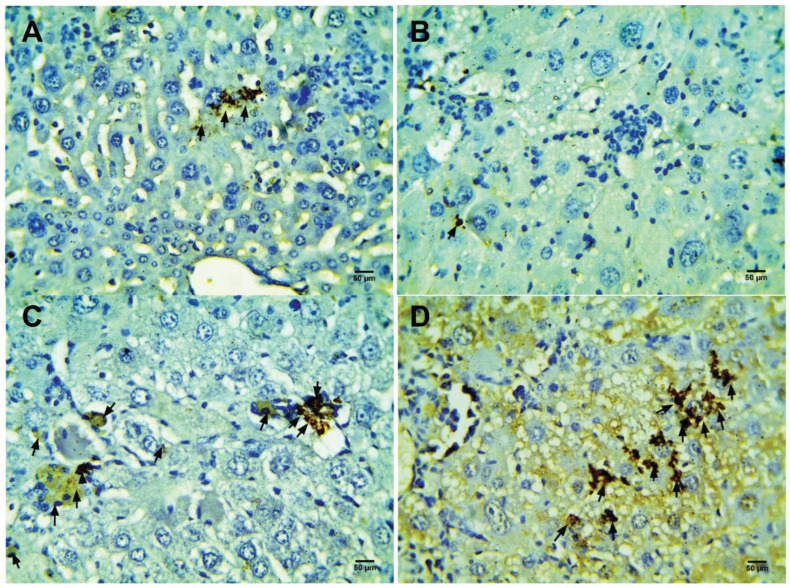
Compound **4d** induced activation of caspase 3 (black arrow). (**A**) Control healthy mice. (**B**) CCl_4_-treated mice. (**C**) CCl_4_-treated mic treated with 50 mg/kg/14 d. (**D**) CCl_4_-treated mice treated with 200 mg/kg/48 h.

P53 is a tumor suppressor and its activation either led to the DNA repair or induction of apoptosis if the DNA damage is extensive [49]. In order to confirm the transactivation of p53 in CCl_4_-treated mice after injection of compound **4d**, the level of apoptotic executioner caspase 3 was detected. The liver sections were immunostained with caspase 3 and the level of staining was examined by bright microscope. Figure 4A revealed that healthy mice showed normal level of caspase 3 (black arrow) while CCl_4_-treated mice showed very low level of caspase 3 (Figure 4B) in comparison to healthy mice. On the other hand, treating mice with compound **4d** at low or high dose (Figure 4C and Figure 4D, respectively) induced the activation of caspase 3, confirming the ability of compound **4d** to activate p53.

### 3.5. Compound ***4d*** Induced Liver Cell Regeneration

**Figure 5:**
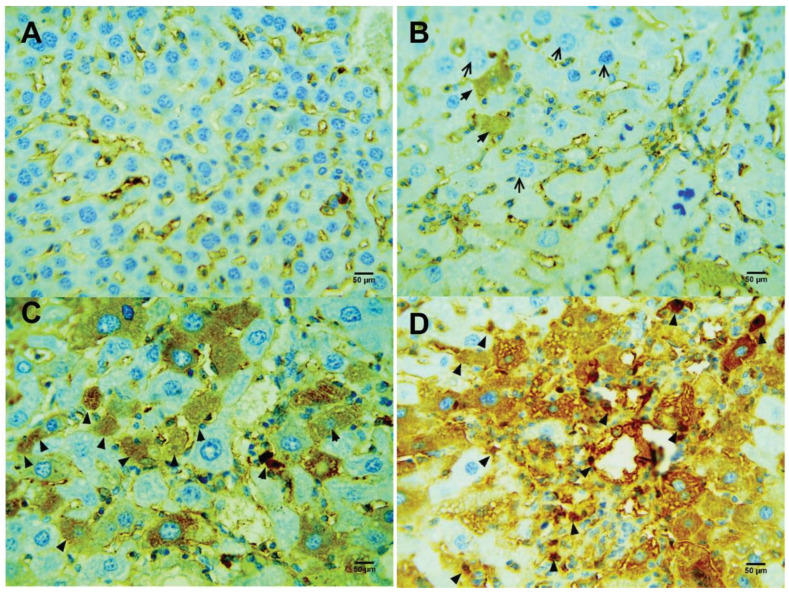
Compound **4d** increased the level of proliferative index Ki-67 (black arrow). (**A**) Control healthy mice. (**B**) CCl_4_-treated mice. (**C**) CCl_4_-treated mic treated with 50 mg/kg/14 d. (**D**) CCl_4_-treated mice treated with 200 mg/kg/48 h.

**Figure 6:**
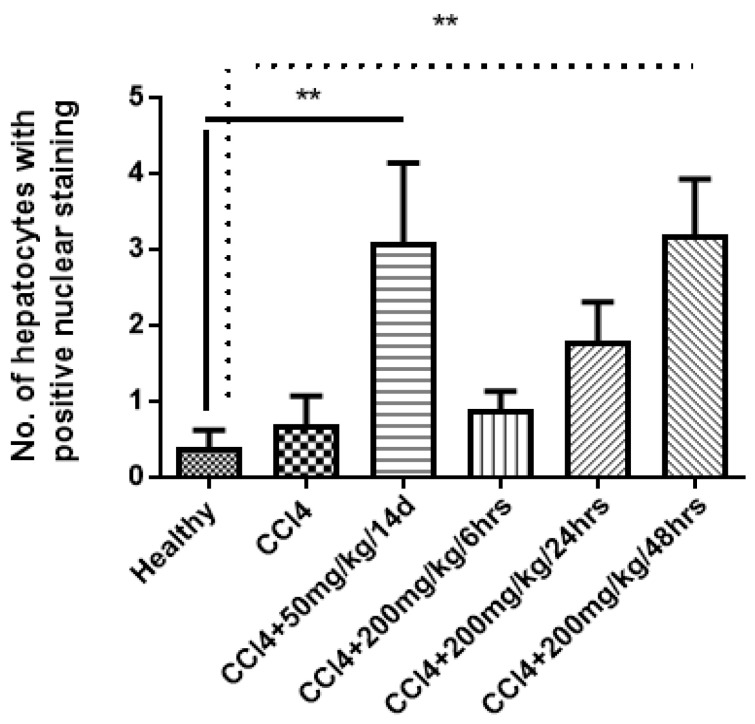
Significant increase in Ki-67 nuclear staining in hepatocytes after treatment of mice with compound **4d**. Statistical analysis was performed by one-way analysis of variance (ANOVA) with Duncan’s multiple comparisons of the means to compare difference between means. ** *p* value < 0.05 is considered significant.

Induction of apoptosis by compound **4d** in liver cells will lead to liver damage and increase the level of enzymes such as ALT and AST. Interestingly, during this study, the level of the two enzymes was decreased close to the normal level in healthy mice, so we hypothesized that compound **4d** might be able to induce liver regeneration after CCl_4_-inducing damage. In order to test this hypothesis, the level of proliferative Ki-67 marker was evaluated using immunohistochemistry. Ki-67 detection in hepatocytes was examined. Healthy mice did not show any hepatocyte Ki-67 staining (Figure 5A), while few hepatocytes were stained (black arrow) with Ki-67 in CCl_4_-treated group (Figure 5B and Figure 6). Treatment of CCl_4_-induced liver damage and treatment of mice with compound **4d** either with low (daily dose for 14 days) or high dose (single dose) significantly increased the number of Ki-67 nuclear stained hepatocytes (Figure 5C and Figure 5D, respectively). Collectively, the presented data indicate that compound **4d** not just stopped the progression of liver cancer but also induced the hepatocyte regeneration.

### 3.6. Preparation of ***4d***-Loaded Proniosomes

**Figure 7:**
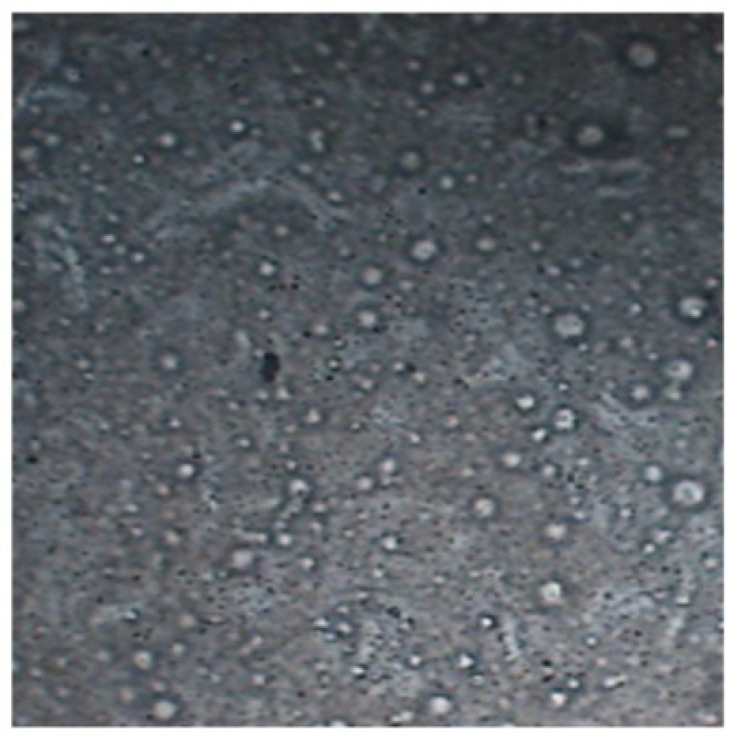
Optical photomicrograph of **4d**-loaded proniosomes after hydration at 25 °C with magnification of (40×).

In the preliminary studies, the amount and type of the selected ingredients were carefully selected based on insights gained from previous studies [35,37,40,44]. The first step in the prescreening of the **4d**-loaded proniosomes is selecting the surfactant that could formulate proniosomes that are rehydrated at 25 °C. It was observed that vesicles were formed on hydration of the Sorbitan monooleate-based proniosomes at 25 °C (Figure 7), whereas the proniosomes containing Sorbitan monostearate required a higher temperature (>60 °C) for formation of the vesicles. Therefore, sorbitan monostearate was not selected for preparing the **4d**-loaded proniosomes due to its high hydration temperature that is not acceptable in the clinical setting.

In addition, the time of hydration was studied to choose the proper conditions for hydration of proniosomes. Table 1 demonstrates that the time of hydration has no significant effect (*p* > 0.05) on %EE. Therefore, a 2 min hydration time was chosen for further studies because a short hydration time would improve patient acceptability.

### 3.7. Fabrication of ***4d***-Loaded Proniosomes According to 2^3^ Factorial Design Using the Slurry Method

**4d**-loaded proniosomes were fabricated using the slurry method. Maltodextrin and mannitol were used as the coating carriers. Sorbitan monooleate was selected as the non-ionic surfactant, and CHOL was added as a membrane stabilizer of the nanovesicles (Table 2).

### 3.8. Preparation of Niosomes Derived from ***4d***-Loaded Proniosomes

**4d**-loaded niosomes were directly formed by hydration of the proniosome powder with distilled water at 25 ± 2 °C. Vesicular structures were formed over the carrier surface due to lipid bilayer swelling that transformed with gentle agitation into multilamellar nanovesicles. The multi-lamellar vesicles are additionally converted by sonication to unilamellar niosomal vesicles [50].

### 3.9. Analysis of 2^3^ Factorial Design

The optimization process aims to determine the levels of variables required for manufacturing high quality formulations. A 2^3^ factorial design was used for the optimization of **4d**-loaded proniosomes. Three independent variables were selected: Sorbitan monooleate to CHOL ratio (X1), the carrier type (X2), and the volume of hydration (X3) (Table 2). The selection of the optimized proniosomal formula was based on attaining maximum EE% (Y1) and maximum Q_12h_ (Y2).

It was observed that the values of R^2^, the predicted R^2^, and the adjusted R^2^ for both the EE % (Y1) and Q_12h_ (Y2) are relatively high, suggesting that the data obtained are highly statistically valid (Table 3).

ANOVA results demonstrated the significance of different factors. The null hypothesis (H0) is rejected, and the alternative hypothesis is accepted when the *p*-value is less than 0.05 (Table 4).

### 3.10. The Effect of Formulation Variables on EE% of ***4d***-Loaded Proniosomes

The EE% of **4d**-loaded proniosomes ranged between 65.64 ± 1.34 to 82.06 ± 2.31 (Table 2). The final Equation of the EE% of **4d**-loaded proniosomes could be expressed in terms of coded factors as follows:

Y1 = + 73.84 + 0.22 × A − 3.09 × B − 5.12 × C

**Figure 8:**
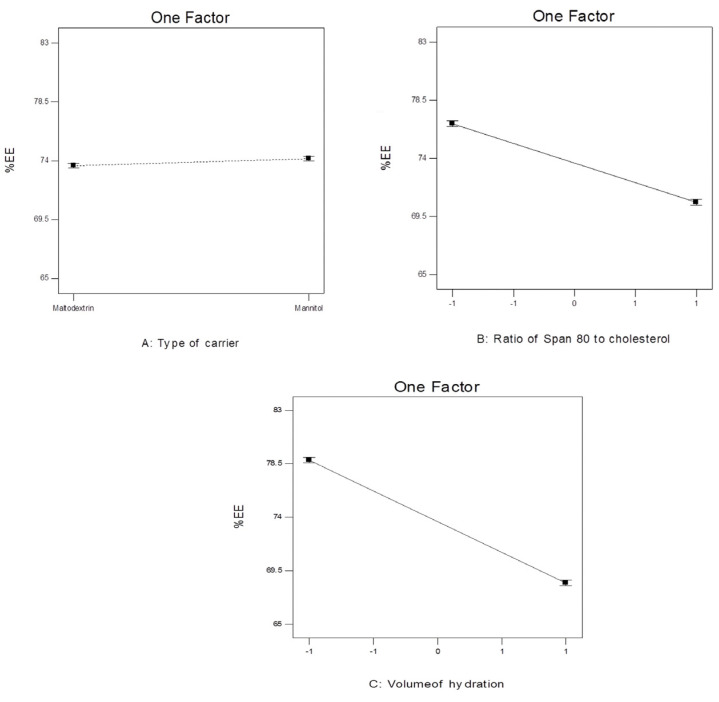
The effect of different independent variables on EE% of **4d**-loaded proniosomes.

Figure 8 demonstrates the effect of different independent variables on the EE% of **4d**-loaded proniosomes. The statistical analysis (Table 4) revealed that both the CHOL-to-Sorbitan monooleate ratio (X1) and the volume of hydration (X3) had a significant impact on EE% (*p* < 0.0001). With respect to the type of carrier (X2), non-significant results (*p* > 0.05) were observed between different formulations that have different carriers.

The drug content of the proniosome-derived niosomal dispersions (entrapped + un-entrapped) was found to be in the range of 94.77 ± 1.59 to 99.55 ± 1.74%. The DLC was found to be in the range of 18.39 ± 0.4 to 49.69 ± 0.9. There was a direct correlation between EE% and DLC of **4d**-loaded proniosomes. DLC is a crucial parameter in the formulation of sustained-release drug delivery systems because it is used to quantify how much drug has been successfully entrapped into a carrier-based delivery system.

### 3.11. The Effect of Formulation Variables on Q_12h_ of ***4d***-Loaded Proniosomes

**Figure 9:**
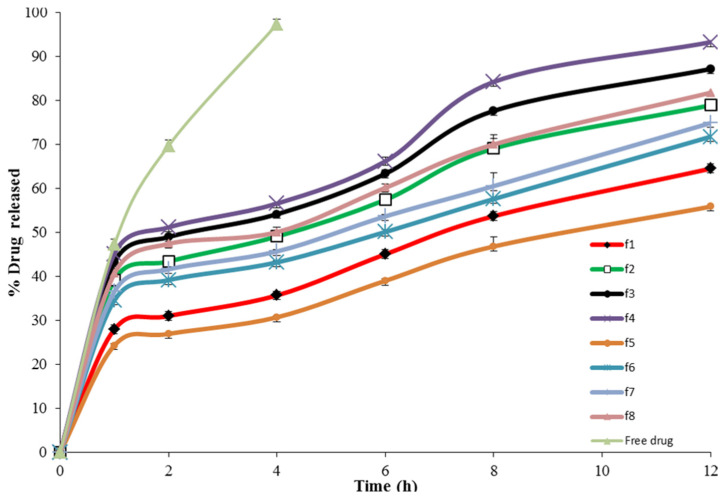
In vitro release of **4d**-loaded proniosomes.

**Figure 10:**
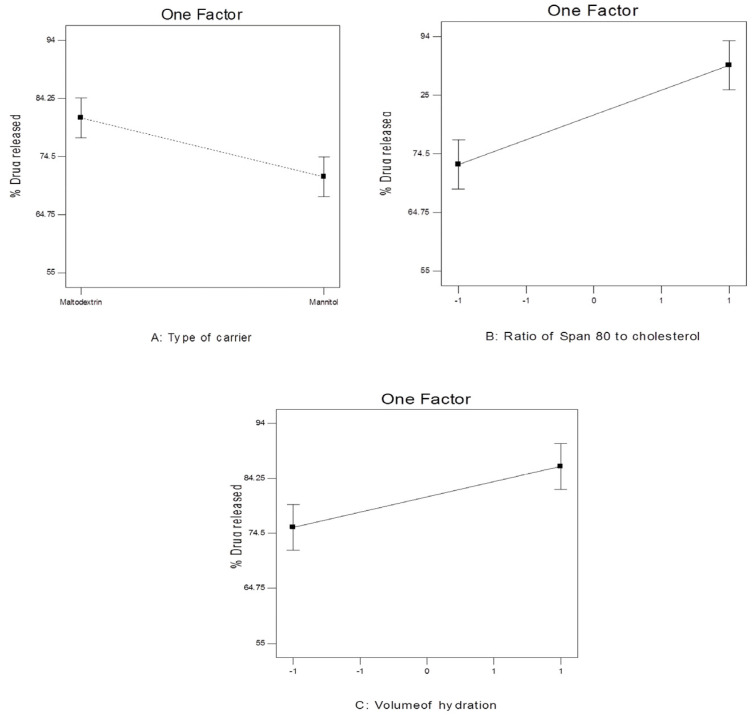
The effect of different independent variables on Q_12h_ of **4d**-loaded proniosomes.

Figure 9 displays the in vitro release profile of **4d** from different proniosomal formulations. Q_12h_ was in the range of 55.87 ± 1.84 to 93.26 ± 1.48. The in vitro release study revealed that the drug release from the proniosomal formulations was slower than that of plain drug that achieved 97.34 ± 1.62% cumulative drug release after 4 h. That confirmed the sustained effect of the proniosomal vesicles [51]. Figure 10 demonstrates the effect of different independent variables on Q_12h_ (Y2).

The final Equation of the in vitro release of **4d**-loaded proniosomes could be expressed in terms of coded factors as follows:

Y2 = + 76.02 − 4.95 × A + 8.25 × B + 5.41 × C

ANOVA results showed that different independent variables had a significant effect on the Q_12h_ of the prepared proniosomes (Table 4). Q_12h_ of **4d**-loaded proniosomes was significantly (*p* < 0.05) related to the type of carrier (X1). The results reveal that Q_12h_ in case of maltodextrin-based proniosomes was higher than that from mannitol-based proniosomes. In addition, the CHOL-to-Sorbitan monooleate ratio (X2) had a significant negative impact (*p* < 0.01) on Q_12h_. However, the volume of hydration (X3) had a significant positive effect (*p* < 0.05) on Q_12h_. The kinetic study showed that the highest correlation coefficient values (R^2^) were for the zero-order model (Table 5).

**Figure 11:**
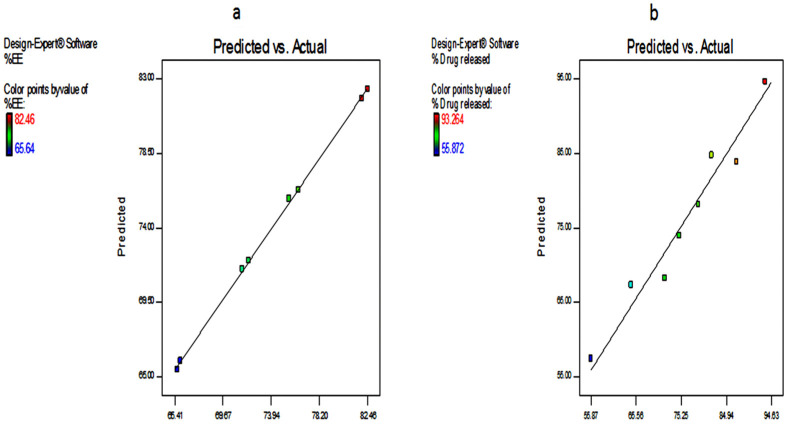
Linearity plots of **4d**-loaded proniosomes shown as observed versus predicted values (**a**) Y1 and (**b**) Y2.

The linear correlation plots between the observed and the predicted values of EE% (Y1) and Q_12h_ (Y2) of **4d**-loaded proniosomes were demonstrated in Figure 11. There is an excellent fit between the predicted and observed values of both Y1 and Y2 in the present model.

### 3.12. Optimization of the ***4d***-Loaded Proniosomes

**Figure 12:**
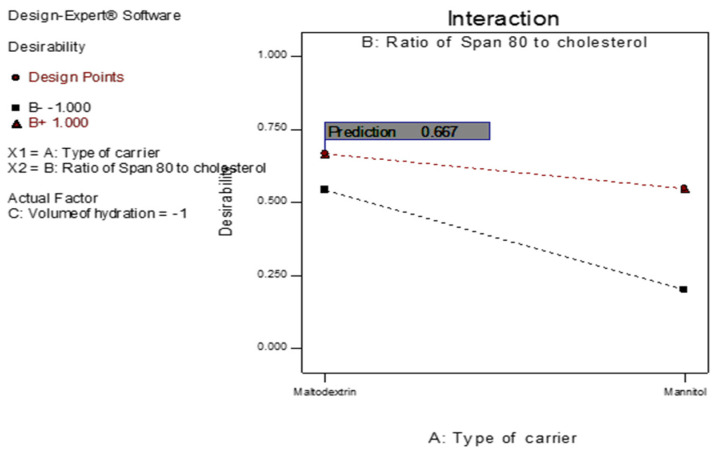
The overall desirability of the **4d**-loaded proniosomes as a function of independent variables.

Optimization of the **4d**-loaded proniosomes was performed on the criteria of attaining maximum EE% and maximum %drug released through numerical analysis using Design-Expert software. The choice of the optimized formula was based on the desirability criteria [52]. F3 had the highest desirability index (0.670); thus, it was selected as the optimized proniosomal formula (Figure 12).

### 3.13. Comparative Study of the Optimized Proniosomal Formula and the Conventional Niosomes

#### 3.13.1. Determination of Entrapment Efficiency (EE%)

EE% of the conventional niosomal formula was found to be 77.06 ± 1.33%. No significant difference (*p* > 0.05) was detected between the optimized proniosomal formula (F3) and the corresponding niosomes.

#### 3.13.2. In Vitro Release Study

**Figure 13:**
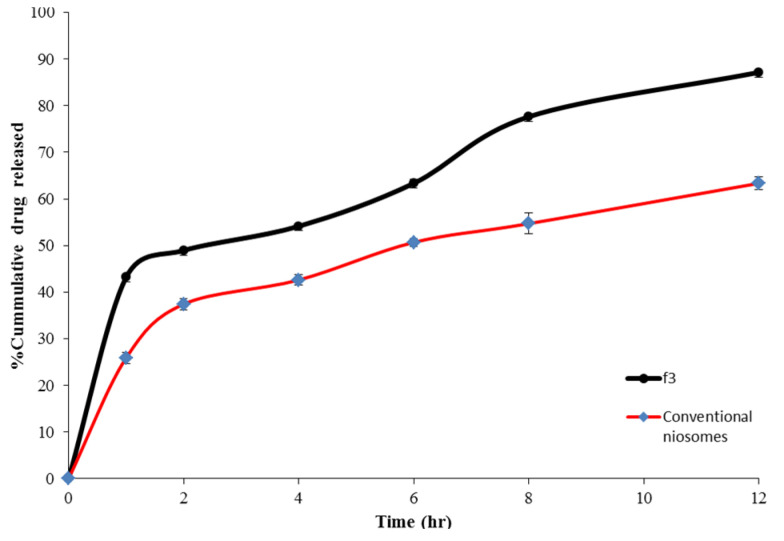
In vitro release profile of the optimized proniosomal formula and the conventional niosomes.

Comparing the in vitro drug release from the optimized proniosomal formula (F3) with that from the corresponding niosomes (Figure 13), it was found that **4d**-loaded proniosomes presented significantly higher cumulative %drug release (*p* < 0.001) than that from the corresponding niosomes that achieved 63.36 ± 1.74% cumulative drug released after 12 h. The in vitro release profile of the optimized proniosomal formula was compared with that of the conventional niosomal formula using the similarity factor test. The estimated values of f_2_ were found to be 34 (less than 50). Therefore, it is obvious that there is a significant difference in the in vitro drug release between the optimized proniosomal formula in comparison to the conventional niosomal formula.

#### 3.13.3. Stability Study

The storage stability of the optimized proniosomal formula and the corresponding niosomal formula was studied for three months at 4–8 °C (Table 6). There was no significant difference in both the drug content and EE% of the stored proniosomal formula (F3) when compared with the fresh proniosomal formula (*p* > 0.05). However, there was a significant decrease in drug content (*p* < 0.01) and EE% (*p* < 0.05) of the fresh niosomal formula when compared to the fresh formula.

### 3.14. Characterization of the Optimized Proniosomal Formula

#### 3.14.1. Micromeritic Properties

It was observed that the angle of repose of **4d**-loaded proniosomal powder (34.22° ± 0.37°) was lower than that of maltodextrin (47.15° ± 0.48°). Therefore, the flowability of the optimized proniosomal powder (F3) is better than that of maltodextrin powder.

#### 3.14.2. Scanning Electron Microscopy (SEM)

**Figure 14:**
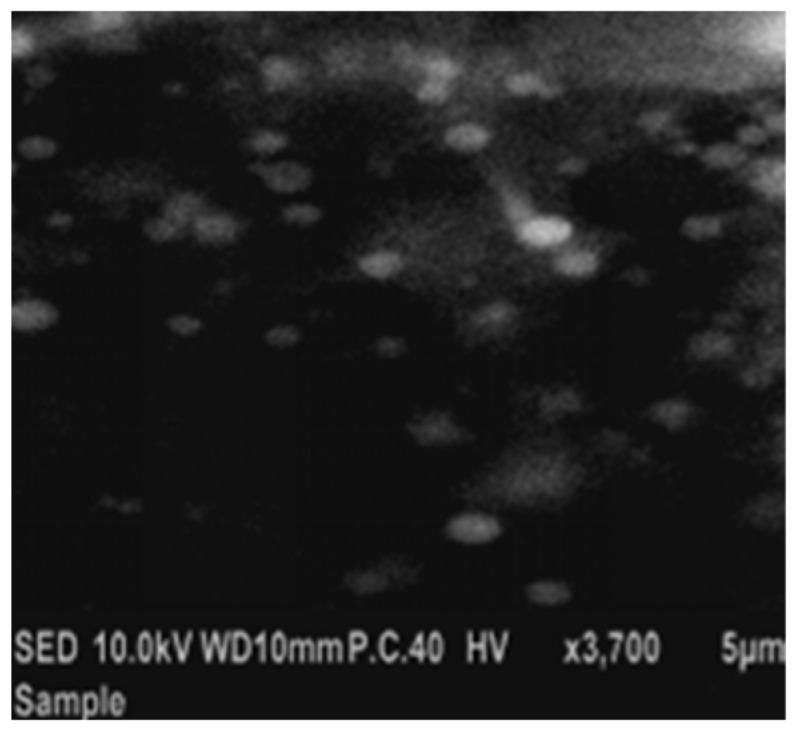
Scanning electron micrograph of the optimized **4d**-loaded proniosomal formula (F3).

The SEM micrograph of the niosomal vesicles formed by hydration of the optimized proniosomal formula (F3) is shown in Figure 14. The **4d**-loaded vesicles appeared as spherical vesicles with sharp boundaries.

#### 3.14.3. Vesicle Size and Zeta Potential Determination

**Figure 15:**
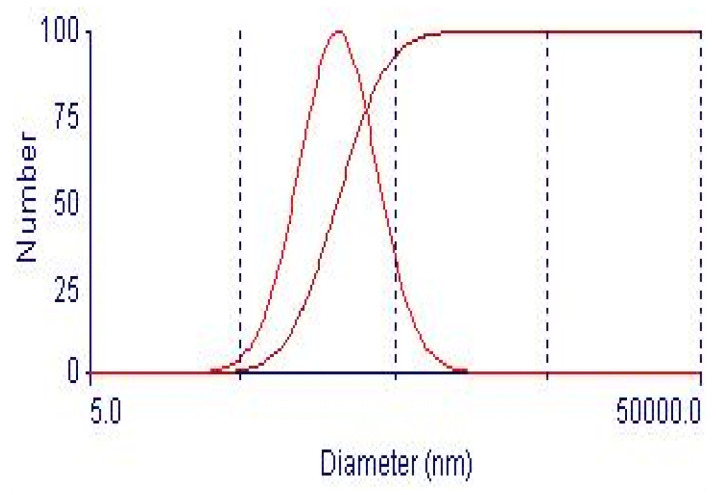
Particle size distribution curve of the optimized **4d**-loaded proniosomal formula (F3).

The particle size distribution of the optimized **4d**-loaded proniosomal formula (F3) exhibited a unimodal symmetric frequency distribution pattern (Figure 15). The mean vesicle size of the hydrated proniosomal formula was 252.3 nm. Moreover, the polydispersity index (PDI) of F3 was low (0.425), demonstrating a homogenous vesicle size distribution.

**Figure 16:**
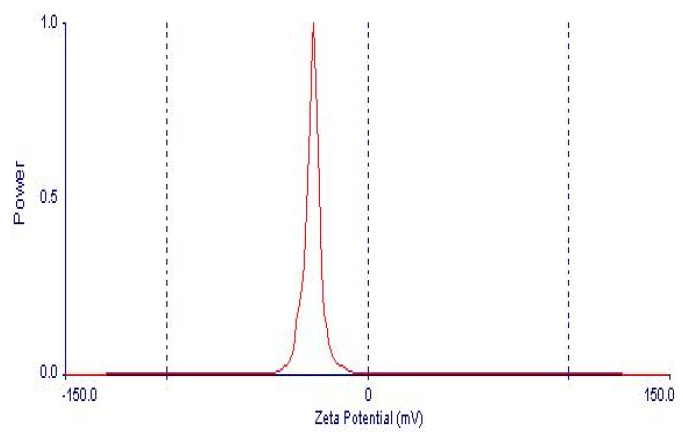
Zeta potential distribution of **4d**-loaded proniosomal formula (F3).

Zeta potential is a measure of the net charge of the colloidal dispersion. Large positive or negative zeta potential exhibits the stability of colloidal dispersions due to repulsion between different vesicles that prevents their agglomeration and provides a stable and uniformly distributed suspension [53]. The optimized **4d**-loaded proniosomal formula has a high zeta potential value (−25.24 mv), showing that this formula is stable (Figure 16).

#### 3.14.4. Fourier Transform Infrared Spectroscopy (FTIR)

**Figure 17:**
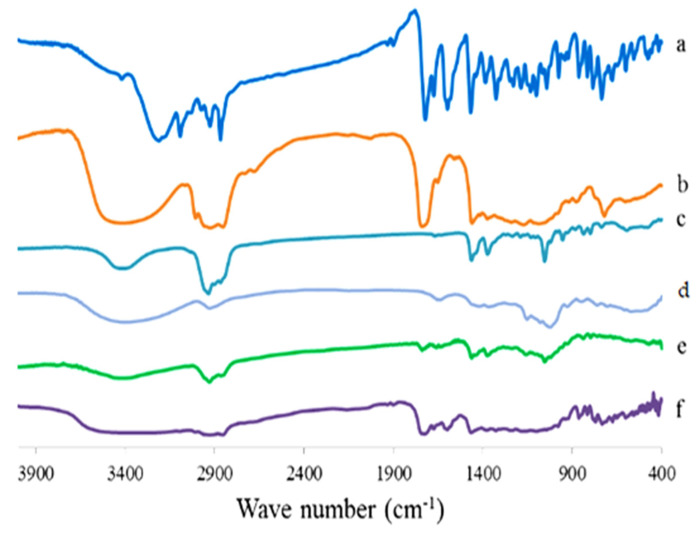
FTIR spectrum of **4d** compound (a), Sorbitan monooleate (b), CHOL (c), maltodextrin (d), plain proniosomes (e), and the optimized **4d** proniosomal formula (F3) (f).

The FTIR spectra of **4d**, Sorbitan monooleate, CHOL, maltodextrin, drug-free proniosomes, and the selected proniosomal formula (F3) were depicted in Figure 17. The FTIR spectrum of **4d** exhibited characteristic peaks at 3188 (NH), 3084 (ArH), 1714 (C=O), and 1670 (CONH) [8]. Sorbitan monooleate displayed characteristic peaks at 3390 cm^−1^, attributed to aliphatic O–H stretch. The absorption bands observed at 2900 and 2831 cm^−1^ are assigned to (C–H stretching), while the peak at 1710 cm^−1^ represents the C=O stretch of ester [53]. Characteristic peaks of CHOL were observed at 3400 cm^−1^, attributed to OH stretching. The bands observed between 2800 and 3000 cm^−1^ correspond to asymmetric and symmetric stretching vibrations of CH_2_ and CH_3_ groups [54]. The FTIR spectrum of maltodextrin exhibited an absorption band at 3399 cm^−1^, corresponding to the OH group, along with a strong broad band observed between 980 cm^−1^ and 1200 cm^−1^, which is a characteristic feature of polysaccharides. Peaks observed at 1152 cm^−1^ and 1018 cm^–1^ were attributed to C–O stretching [55]. The IR spectrum of plain proniosomes displayed characteristic peaks of CHOL, Sorbitan monooleate, and maltodextrin, albeit with reduced intensity. In the FTIR spectrum of the chosen proniosomal formula, characteristic peaks of **4d** were observed with reduced intensity and minor shifting.

#### 3.14.5. Differential Scanning Calorimetry (DSC) Study

**Figure 18:**
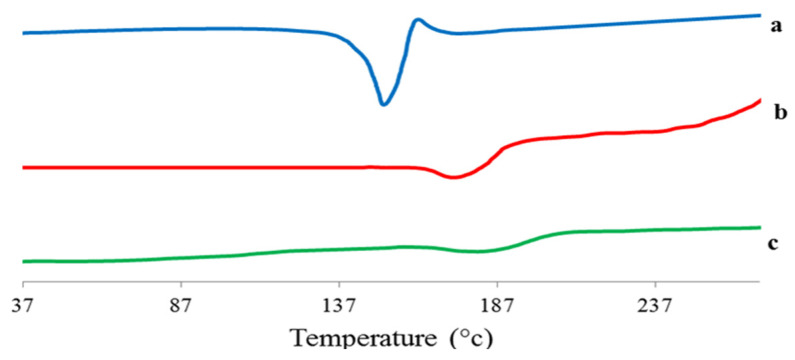
DSC thermogram of **4d** (a), plain proniosomes (b), and the optimized **4d**–loaded proniosomal formula (F3) (c).

Figure 18 presents the DSC thermograms of the **4d** compound, plain (drug-free) proniosomes, and the selected proniosomal formula. The DSC thermogram of **4d** exhibited a sharp endothermic peak at 150 °C, corresponding to its melting point, indicating the crystallinity of **4d [8]**. The plain proniosomes demonstrated the appearance of a broad endothermic peak at 172.3 °C. The optimized proniosomal formula (F3) showed the absence of the characteristic endothermic peak of **4d** and a shift of the endothermic peak of the lipid bilayer to 181.5 °C.

## 4. Discussion

### 4.1. In Vivo Study of ***4d***

Thrombocytopenia is considered as a drawback for the clinical utility of most MDM2-P53 inhibitors. Therefore, in this study, the efficacy and safety of new spirooxindole derivate has been investigated in Balb/c mice. The results revealed the safety of either low or high dose of compound **4d** in the presented animal model. This finding needs to be evaluated in other animal models, such as rats and monkeys^17^, and also the repeated cycles of dosing may led to a complete recovery of damaged liver cells. Histopathology investigation showed that compound **4d** recovered the damaged liver cells and stopped the mitosis (lower mitotic index in comparison to untreated mice), and this observation may be explained by the ability of compound **4d** to stop the cell growth at G2/M phase of cancer cells in the in vitro study [7]. The ability of compound **4d** to activate p53 was also evaluated, and the immunohistochemistry showed the activated p53 staining in the liver sections. Activated p53 has been linked to the induction of apoptosis and an increase in caspase 3 [1,48,56,57]. Here, in this study, the activated p53 by compound **4d** led to the higher detection of activated caspase 3 in the liver sections. These data are in agreement with the in vitro study where compound **4d** killed the cancer cells via the activation of caspase 9 and caspase 8, leading to the activation of caspase 3. In the same time, the activated p53 increased the level of Ki-67 proliferative marker. Ki-67 is a nuclear protein that is linked to the proliferating cells. Its mRNA level is highly increased during all cell cycle phases even in the quiescent cells [58,59,60]. Therefore, it is applied in routine diagnostic marker for tumor cells in order to determine the prognosis of the patients. However, Ki-67 is not only linked to the cancer proliferation but also its expression is increased after partial hepatectomy [61]. In the current study, liver dysplasia was reached after treatment of mice with CCl_4_, leading to the damage of liver cells. Cell proliferation is a biological process in response to different stimuli, such as infection of injury [62]. In response to the injection of CCl_4_ in mice followed by treating these mice with compound **4d**, the p53, caspase 3, and Ki-67 were activated and increased in order to stop the proliferation of premalignant cancer cells and, at the same time, to regenerate the damaged hepatocytes.

### 4.2. Molecular Modeling

**Figure 19:**
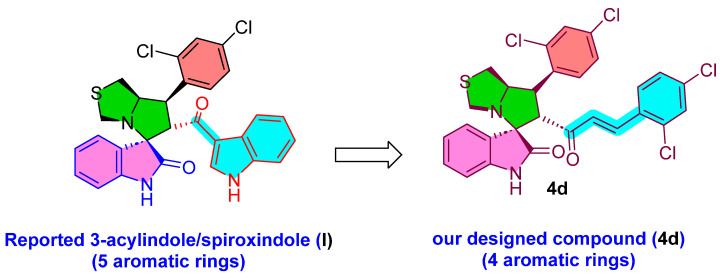
Designed the compound **4d**.

In continuation of our recent work for synthesis of new spirooxindoles tethered with 3-acylindoles as potent anti-apoptotic agents with high safety profile, our aims were direct, to synthesize new analogs with high potency, high safety, and with good ADME properties, such as lipophilicity. In this regard, compound **4d** was constructed by replacement of aromatic heterocyclic ring (3-acylindole) by un-saturated aryl. The aryl moiety was selected as the same aryl substituent attached to pyrrolidine ring. The idea was to keep the geometry with reduction in ring numbers within the whole structure of molecules (Figure 19).

#### 4.2.1. Prediction of Ligand Efficiency (LE) and Ligand Lipophilic Efficiency (LLE)

##### Ligand Efficiency (LE) Scores: 12

Ligand Efficiency (LE) is employed to gauge the effectiveness of compounds and ascertain binding affinity (in terms of binding energy or pIC_50_) relative to the number of heavy atoms present in a molecule. This methodology offers a means to compare the affinity of molecules while accounting for differences in their size [13].

LE is the ratio of the affinity of a ligand divided by the number of heavy (non-hydrogen) atoms in the molecule (LE = ΔG/[number of heavy atoms. LE can be calculated according to the following Equation:

LE = (pIC_50_ × 1.37)/NHA.

where:

IC_50_ = half-maximal inhibitory concentration (in terms of molar concentration).

NHA = non-hydrogen atom. The recommended LE value for lead-likeness should be in the range of 0.3. The acceptable LE value for drug-likeness should be higher than 0.3.

From Table 7, LE values of compound **4d** equal 0.2 among all tested cell lines which means yet this compound has LE. Then, we directed to measure LEE as it correlated to the LogP of compound.

##### Ligand Lipophilic Efficiency (LLE) 14

Enhancing compound activity without concurrent increases in lipophilicity presents a significant challenge. The lipophilic ligand efficiency (LLE) offers a method to evaluate a compound’s affinity while considering its lipophilicity (the disparity between potency and log P). Hence, LLE serves as a robust and pragmatic approach to regulate lipophilicity, thereby averting potential “molecular obesity” during the optimization process.

It can be calculated as follows:

LLE = pIC_50_ − Clog P

LLE value ≥ 3 for lead compound is acceptable and for drug like candidate LLE value ≥ 5 is recommended. To obtain more accurate results, log P was calculated practically, and we measure Log K as an indicator for log P and can be used instead of it [15,16,17].

Log K = 1.19: LLE values for our compound are illustrated in Table 7 and it should good value (4.50–4.88).

Based on this value, our compound has a good LEE value, and in addition to its safety profile (high SI), this compound is a promising anticancer drug candidate.

#### 4.2.2. Shape Alignment and Scoring Using ROCS

The alignment quality between the database (consisting of reported spirooxindoles) and the query molecule (compound **4d**) was assessed using the Tanimoto Combo score, which is the sum of the Shape Tanimoto and Color Tanimoto scores. The Shape Tanimoto score reflects the shared volume and mismatch volume between molecules, ranging from 0 to 1.0. Meanwhile, the Color Tanimoto score indicates the degree of matching or mismatching of light chemical features in three dimensions, also ranging from 0 to 1.0. Both the query and database molecules were amalgamated into a single species by fragment disconnected non-chemically, meaning pieces of a molecule were combined. The potent anticancer compound **I** showed highest similarity to compound **4d** with Tanimoto Combo equaling 1.07.

**Figure 20:**
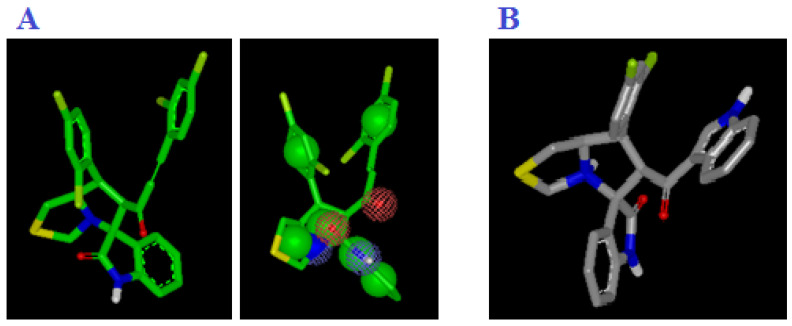
(**A**) Representation shape and color atoms of compound **4d** by vROCS application; (**B**) representation shape and color atoms of compound **I** vROCS application.

**Figure 21:**
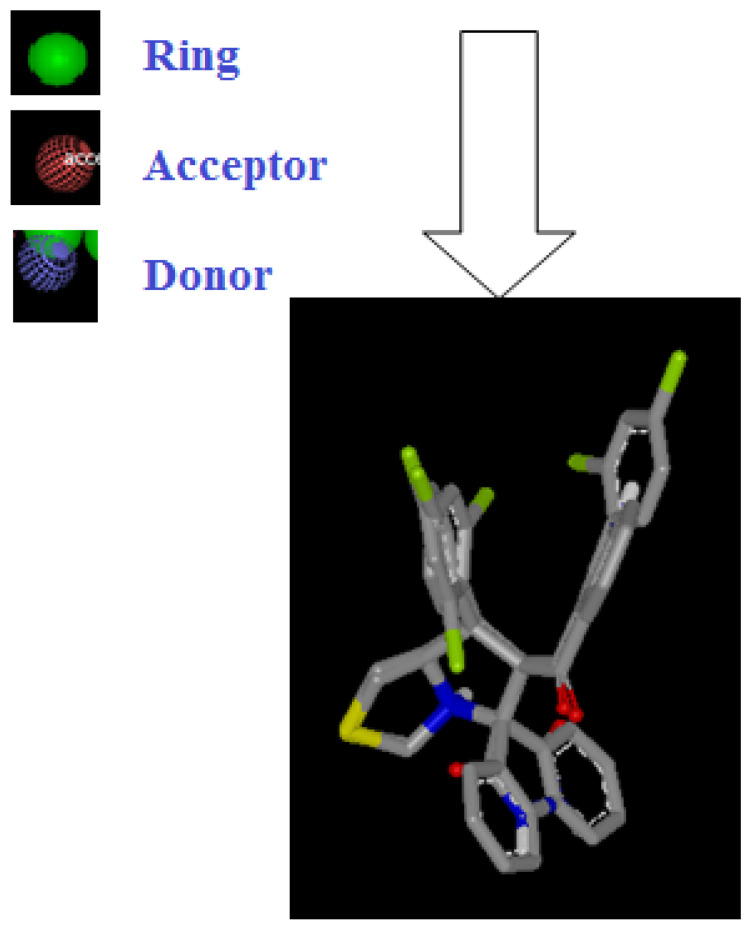
Representation shape of compound **4d** overlay compound **I** vROCS application.

The ROCS shape and color analyses for our compound (**4d**) as a query model showed a shape volume with 4 rings, 2 donors, and 2 acceptors (Figure 20A), and the reported compound **I** adopted the same shape with 5 rings, 2 donors, and 2 acceptor species (Figure 20B). The alignment and overlay using ROCS between our compound (query) and database molecule (compound) showed complete alignment with difference in position of spirooxindole (Figure 21).

### 4.3. Preliminary Screening Study of the ***4d***-Loaded Proniosomes

The prescreening study of the **4d**-loaded proniosomes involves selecting the non-ionic surfactant that could formulate proniosomes that are rehydrated at 25 °C and choosing the most appropriate hydration time. Sorbitan monooleate-based proniosomes could be rehydrated at 25 °C, whereas the sorbitan monostearate-based proniosomes required a higher temperature (>60 °C). Therefore, Sorbitan monooleate was chosen as the non-ionic surfactant. This selection can be rationalized by the fact that vesicle formation typically occurs at a temperature higher than the phase transition temperature of the non-ionic surfactant. Sorbitan monostearate has a phase transition temperature of 53 °C, whereas Sorbitan monooleate has a phase transition temperature of −12 °C. The lower gel-to-liquid phase transition temperature of Sorbitan monooleate may be attributed to the presence of an unsaturated double bond in the oleate chain [50].

Furthermore, 2 min hydration time was chosen because a short hydration time would improve the applicability of the formulation, and increasing the hydration time had no significant effect (*p* > 0.05) on %EE. These results agreed with Pawar et al., who found that there was no significant effect of the time of hydration on the EE% of the drug in niosomes [63]. That is also in accordance with other researchers, such as Ramkanth et al. [64] and Patil et al. [65], who performed hydration of proniosomes for 2 min. Moreover, Sammour et al. [36] reported that increasing the time of hydration has a significant negative impact (*p* < 0.05) on %EE. However, other researchers reported that as the hydration time increased, more of the drug would be entrapped [66].

### 4.4. Fabrication of ***4d***-Loaded Proniosomes and Analysis of 2^3^ Factorial Design

A 2^3^ factorial design was used for the optimization of **4d**-loaded proniosomes. The high values of R^2^, adjusted R^2^, and predicted R^2^ for both the EE% (Y1) and Q_12h_ (Y2) reflect that the data obtained are statistically valid. The adjusted R^2^ is often regarded as more reliable than the R2 value because it adjusts for the addition of variables to the model. When additional independent variables are incorporated into the model, the R^2^ value tends to increase. Conversely, the adjusted R^2^ value accounts for whether the additional variables enhance or diminish the explanatory power of the model. As a result, the adjusted R^2^ value may be equal to or lower than the R^2^ value. In the current model, for both EE% (Y1) and Q_12h_ (Y2), the adjusted R^2^ value was observed to be lower than the R^2^ value. Predicted R^2^ values assess the predictability of the response value within the model. As depicted in Table 3, the adjusted and predicted R^2^ values for both Y1 and Y2 exhibited reasonable agreement, with the difference between their values being less than 0.2. Adequate precision, which determines the signal-to-noise ratio, was found to exceed the desired value (4) for both Q_12h_ and EE%. Consequently, this model is deemed suitable for exploring the design space [67,68].

### 4.5. The Effect of Formulation Variables on EE% of ***4d***-Loaded Proniosomes

High EE% of **4d**-loaded proniosomes values could be attributable to the poor water solubility of **4d** and its dissolution in the nanovesicle lipid bilayers [69]. It is obvious that CHOL plays a vital role in the encapsulation of drug within the vesicles. Increasing the CHOL concentration has a positive impact on the EE% (*p* < 0.05). That may be attributed to the fact that the CHOL acts as the vesicular cement that forms more rigid and less leaky vesicles and hence increases the EE% [66]. Concerning the volume of hydration (X3), it is obvious that X3 had a significant negative effect on %EE. That could be attributable to the fact that changing the surfactant-to-water ratio during the hydration step might affect the microstructure and the rigidity of the nanovesicles [70]. Moreover, Sammour et al. [36] reported that CHOL has an amphiphilic nature and orients its carbon chain towards the surfactant’s hydrocarbon chain and its hydrophilic OH group towards the aqueous phase. The nanovesicles could, therefore, be formed with proper rigidity by alternative location of the rigid steroidal skeleton with surfactant molecules in the bilayer and by restricting the movement of hydrocarbon carbons; the large volume of the aqueous hydrating medium used could decrease the rigidity and encapsulation of the vesicles formed.

### 4.6. The Effect of Formulation Variables on Q_12h_ of ***4d***-Loaded Proniosomes

The rapid in vitro drug release observed in the initial hours for **4d**-loaded proniosomes may be attributed to the release of adsorbed drug from the lipophilic region of nanovesicles. This phenomenon could facilitate achieving the optimal loading dose.

The Q_12h_ of **4d**-loaded proniosomes, particularly in the case of maltodextrin-based proniosomes, exhibited a significantly higher value (*p* < 0.05) compared to that from mannitol-based proniosomes. This difference could be ascribed to the higher solubility of maltodextrin [37].

Furthermore, it is evident that Q_12h_ significantly increased (*p* < 0.01) as the concentration of CHOL decreased. This observation may be attributed to the mechanism of in vitro drug release from the niosomal vesicles being influenced by the rigidity of the membrane. As the concentration of CHOL increases, drug efflux decreases due to its membrane stabilizing ability, which involves filling the pores in the vesicular bilayer, resulting in sustained drug release [40]. Regarding the volume of hydration, it is apparent that it had a significant positive effect (*p* < 0.05) on Q_12h_. This could be attributed to the increased drug leakage [66].

### 4.7. Optimization of the ***4d***-Loaded Proniosomes

The optimization of the **4d**-loaded proniosomes was conducted through numerical analysis using Design-Expert software. Formula F3 was chosen as the optimized **4d**-loaded proniosomal formulation based on the criteria of achieving maximum EE% and maximum % of drug released.

### 4.8. Comparative Study of the Optimized Proniosomal Formula and the Conventional Niosomes

There was no significant difference in the EE% (*p* > 0.05) between the optimized proniosomal formula (F3) and the corresponding niosomes. However, the cumulative % drug release of **4d**-loaded proniosomes was significantly higher (*p* < 0.001) than that from the corresponding niosomes after 12 h. This difference may be attributed to the adsorption of the lipid coat of proniosomal vesicles on maltodextrin, thereby increasing its effective surface area [31]. Moreover, it could be attributed to the enhancement of solubility of **4d** and change in its structure from the crystalline to amorphous state in the proniosomal vesicles [40]. Hence, proniosomes provide sustained-release carriers with higher dissolution profiles than the corresponding niosomes.

In addition, the stability of **4d**-loaded proniosomes, after storage for 3 months at 4–8 °C, was significantly higher than the corresponding niosomes, indicating that proniosomes offer a stable system that could overcome the storage problems associated with conventional niosomes.

### 4.9. Characterization of the Optimized Proniosomal Formula

Good flowability of powders is a key property of solid dosage forms. Flowability is described by the angle of repose (θ). A value of θ between 31 and 35° describes good flowability of powder and a value between 46 and 55° describes poor flowability [40]. Accordingly, F3 is considered to be of good flow properties, while the flowability of pure maltodextrin powder is rated as poor flow. Hence, the flowability of the optimized proniosomal powder (F3) is better than that of maltodextrin powder.

The optimized vesicles (F3) appeared as spherical nanovesicles. The spherical shape of **4d**-loaded vesicles may be explained by the fact that Sorbitan monooleate could form a closed bilayered vesicle in the aqueous environment due to its amphoteric nature in addition to the tendency of niosomal vesicles to minimize their surface-free energy [71,72].

The FTIR spectrum of plain (drug-free) proniosomes exhibited characteristic peaks of CHOL, Sorbitan monooleate, and maltodextrin, albeit with reduced intensity, possibly due to the formation of a lipid bilayer. On the other hand, the FTIR spectrum of the selected proniosomal formula (F3) displayed characteristic peaks of **4d**, confirming the absence of any chemical interactions between **4d** and other excipients [73]. The reduced intensity and minor shifting of the characteristic peaks of **4d** can be attributed to the formation of Van der Waals forces, hydrogen bonds, or dipole–dipole interactions between **4d** and other proniosome components. These interactions likely contribute to the formation of stable proniosomal vesicles with high drug entrapment efficiency [54].

DSC could be used to detect the thermal behavior and physical state of drug. DSC thermogram of the plain proniosomes demonstrated the appearance of a broad endothermic that may be attributable to the interaction of different components of proniosome during development of the lipid bilayer [54]. The optimized proniosomal formula (F3) exhibited disappearance of the characteristic endothermic peak of **4d** and a shift of the endothermic peak of the lipid bilayer that may be explained on the basis of changing **4d** structure from crystalline to amorphous state due to perfect encapsulation of **4d** into proniosomes [74]. The presence of **4d** in the amorphous state results in improved dissolution due to the absence of crystalline lattice bonds that need to be broken [75].

## 5. Conclusions

This in vivo study presents a novel, safe, and effective chemical entity of spirooxindole derivatives as a potential selective therapy for several types of cancers. The extensive in vivo studies, including histopathology, immunohistochemistry, and molecular biology, on such an interesting compound proved the potential of this compound for further preclinical and clinical study after carrying out a full study on its bioavailability, pharmakodynamic, and pharmacokinetic activity. The ongoing work is addressing the possible use to halt the progression of liver cancer, metastasis, and portal vein thrombosis, which may lead to death. Moreover, this invention may help the induction of regeneration and recovery of damaged early cancer cells either in liver or other organs and may lead to improving recovery and remission among cancer patients. The present invention also deals with developing an industry-feasible proniosomal drug delivery system of **4d** as drug reservoirs that could achieve controlled drug release. **4d**-loaded proniosomes were prepared as a dry free-flowing powder that can be reconstituted at room temperature to niosomal suspension prior to use. **4d**-loaded proniosomes exhibited high entrapment efficiency in the range of 65.64 ± 1.34 to 82.06 ± 2.31% and could prolong the in vitro drug release rate up to 12 h. The optimized proniosomal formula (F3) showed significantly higher cumulative %drug release and higher stability than the conventional niosomes. FTIR study exhibited the absence of any chemical interactions between **4d** and other excipients. DSC study displayed proper entrapment of **4d** within the nanovesicles and change in its structure from the crystalline to the amorphous state. Thus, the formulation of **4d** compound as proniosomes is considered to be a stable and prolonged drug delivery system.

## 6. Patents

The work reported in this manuscript has been disclosed in the patent no. US11944604B1.

## Figures and Tables

**Table 1:** Prescreening study for formulation of **4d**-loaded proniosomes.

Formula	Time of Hydration (min)	* EE%
P1	2	81.94 ± 1.35
P2	5	82.07 ± 1.79
P3	30	82.04 ± 1.86

Notes: * the values are expressed as mean ± SD (*n* = 3), 1 g of carrier per 1 mmole of total lipid mixture, volume of hydration is 10 mL. Abbreviations: EE, entrapment efficiency.

**Table 2:** Experimental runs, independent variables, and dependent variables in 2^3^ factorial design used for optimization of **4d**-loaded proniosomes.

Formula Code			Variables		
	Independent				Dependent
	X1	X2	X3	Y1 *	Y2 *
F1	−1	−1	−1	81.94 ± 1.35	64.51 ± 1.59
F2	−1	−1	1	71.38 ± 1.24	78.92 ± 1.97
F3 #	−1	1	−1	75.52 ± 1.23	87.16 ± 1.78
F4	−1	1	1	65.64 ± 1.34	93.26 ± 1.48
F5	1	−1	−1	82.06 ± 2.31	55.87 ± 1.84
F6	1	−1	1	71.94 ± 1.33	71.76 ± 2.32
F7	1	1	−1	75.92 ± 1.38	74.88 ± 1.26
F8	1		1	65.93 ± 1.29	81.78 ± 1.42
Independent Variables				Low (−1)	High (+1)
X1: Type of carrier				Maltodextrin	Mannitol
X2: Ratio of Sorbitan monooleate to CHOL (µmolar ratio)				140:60	160:40
X3: Volume of hydration (ml)				10	15

Notes: Y1: EE (%), Y2: Q_12h_ (%), * the values are expressed as mean ± SD (*n* = 3), # Optimized Formula, 1 g of Carrier per 1 mmole of total lipid mixture. Abbreviations: Q_12h_, drug released after 12 h; EE, entrapment efficiency; cholesterol, CHOL.

**Table 3:** Output data of the 2^3^ factorial design of **4d**-loaded proniosomes.

Responses	*R* ^2^	Adjusted *R*^2^	Predicted *R*^2^	Adequate Precision
EE% (Y1)	0.9995	0.9992	0.9981	128.20
Q_12h_ (Y2)	0.9549	0.9211	0.8197	15.52

Abbreviations: R^2^, the coefficient of determination; Q_12h_, % drug released after 12 h; EE, entrapment efficiency.

**Table 4:** ANOVA for the 2^3^ factorial design of **4d**-loaded proniosomes.

Depndent Variable	Source	SS	Df	MS	F-Value	*p*-Value
Y1	Model	279.64	3	93.21	2794	<0.0001
	X1	0.23	1	0.23	7.03	0.0569
	X2	73.87	1	73.87	2214.22	<0.0001
	X3	205.54	1	205.54	6160.74	<0.0001
Y2	Model	974.89	3	324.96	28.24	0.0038
	X1	195.64	1	195.64	17.00	0.0146
	X2	544.86	1	544.86	47.36	0.0023
	X3	234.38	1	234.38	20.37	0.0107

Notes: Y1: EE (%), Y2: Q_12h_ (%), ratio of Sorbitan monooleate to CHOL (X1), the carrier type (X2), the volume of hydration (X3), values of Prob > F″ less than 0.05 indicate that the model terms are significant. Abbreviation: SS, sum of squares; Df, degree of freedom; MS, mean of squares.

**Table 5:** The calculated correlation coefficients for the in vitro release of **4d**-loaded proniosomes employing different kinetic orders.

Formula	Zero Order	First Order	Higuchi Model	Hixson Crowell	Korsmeyer–Pappas
F1	0.9950	−0.9933	0.9809	0.9949	0.9858
F2	0.9925	−0.9897	0.9829	0.9926	0.9855
F3	0.9869	−0.9691	0.9720	0.9806	0.9698
F4	0.9821	−0.9711	0.9726	0.9800	0.9715
F5	0.9936	−0.9933	0.9792	0.9939	0.9866
F6	0.9973	−0.9847	0.9760	0.9905	0.9748
F7	0.9979	−0.9856	0.9798	0.9917	0.9775
F8	0.9939	−0.9805	0.9789	0.9897	0.9768

**Table 6:** Effect of storage on the properties of the optimized proniosomal formula and the corresponding niosomal formula.

Parameter	Proniosomal Formula		Niosomal Formula	
	Fresh	Stored	Fresh	Stored
Drug content (%)	98.85 ± 1.45	96.63 ± 2.18	99.23 ± 1.45	91.84 ± 1.79
EE (%)	75.52 ± 1.23	71.33 ± 1.47	77.06 ± 1.33%	50.13 ± 2.11%

Notes: Each value represents mean ± SD (*n* = 3), optimized **4d**-loaded proniosomes is F3. Abbreviations: EE, entrapment efficiency.

**Table 7:** Summary of ligand efficiency scores for target compound-**4d** to be considered during fragment-based drug discovery (FBDD).

	NNHA	Clog P (log K)	HCT-116			HepG2			PC-3		
			2			0.85			1.8		
			pIC_50_	LE	LLE	IC_50_	LE	LEE	pIC_50_	LE	LEE
Target compound-**4d**	37	1.19	5.69	0.21	4.5	6.07	0.22	4.88	5.74	0.21	4.55

## Data Availability

The original contributions presented in the study are included in the article/supplementary material, further inquiries can be directed to the corresponding authors.

## Supplementary Materials

The following supporting information can be downloaded at https://www.mdpi.com/article/10.3390/pharmaceutics17010093/s1: Table S1: No alteration in hemoglobin, platelets or leukocyte count was observed after treatment of mice with compound **4d**. Statistical analysis was performed, and data were not significant (*p* value > 0.05). Table S2: Biochemical markers of liver function (mean ± SD) was improved after compound **4d** treatment for 24 or 48 h without any side effect on kidney. Figure S1: H&E staining of kidney showing no pathological alteration in glomeruli (G) and tubules (T) of mice treated with (A) 10% DMSO, (B) 50 mg/kg compound **4d**, (C) 200 mg/kg/24 h compound **4d** and (D) 200 mg/kg/48 h compound **4d**. (HE, ×: 100). Figure S2: H&E staining of heart showed no pathological alteration in cardiomyocytes of mice treated with (A)10% DMSO, (B) 50 mg/kg compound **4d**, (C) 200 mg/kg/24 h compound **4d** and (D) 200 mg/kg/48 h compound **4d**. (HE, ×: 100). Figure S3: H&E staining of spleen showed no pathological alteration in red pulp (R) and white pulp (W) in mice treated with (A) 10% DMSO, (B) 50 mg/kg compound **4d**, (C) 200 mg/kg/24 h compound **4d** and (D) 200 mg/kg/48 h Compound **4d**. (HE, ×: 100).
