# Supplementary material for: Nanoformulation of Spirooxindole and Methods for Treating Hepatocellular Carcinoma
Source: Pharmaceutics. 2025 Jan 12;17(1):93. doi: 10.3390/pharmaceutics17010093 (PMC11768502; doi:10.3390/pharmaceutics17010093)
Supplement: Supplementary file 1 [file pharmaceutics-17-00093-s001.zip › pharmaceutics-3245208-supplementary.pdf]

pharmaceutics

Supporting Materials

Nanoformulation of Spirooxindole and Methods for Treating Hepatocellular Carcinoma

Assem Barakat [1,*], Fardous F. El-Senduny [2,3], Mohammad Shahidul Islam [1], Abdullah Mohammed Al-Majid [1], Yaseen A. M. M. Elshaier [4], Eman A. Mazyed [5] and Farid A. Badria [6,*]

1 Department of Chemistry, College of Science, King Saud University, P.O. Box 2455, Riyadh 11451, Saudi Arabia; mislam@ksu.edu.sa (M.S.I.); amajid@ksu.edu.sa (A.M.A.-M.)
2 Department of Pathology & Laboratory Medicine, Sylvester Comprehensive Cancer Center, Miller School of Medicine, Miami, FL 33136, USA; fxe123@med.miami.edu
3 Department of Chemistry, Faculty of Science, Mansoura University, Mansoura 35516, Egypt
4 Department of Organic and Medicinal Chemistry, Faculty of Pharmacy, University of Sadat City, Menoufiya 32958, Egypt; yaseen.elshaier@fop.usc.edu.eg
5 Department of Pharmaceutical Technology, Faculty of Pharmacy, Kaferelsheikh University, Kaferelsheikh, 33516, Egypt; eman_mazyad@pharm.kfs.edu.eg
6 Department of Pharmacognosy, Faculty of Pharmacy, Mansoura University, Mansoura 35516, Egypt
* Correspondence: ambarakat@ksu.edu.sa (A.B.); faridbadria@gmail.com (F.A.B.)

Table S1: No alteration in hemoglobin, platelets or leukocyte count was observed after treatment of mice with compound **4d**. Statistical analysis was performed, and data was not significant (P value > 0.05).

Parameters	DMSO[a]	50 mg/kg/14d[b]	200 mg/kg/24hrs[c]	200 mg/kg/48hrs[d]	*P*
**Hb**	11.7±1.6	10.16±1.1	10.1±1.43	10.4±1.78	a *vs* b = 0.133<br>a *vs* c =0.122<br>a *vs* d = 0.260
**Platelets**	749.2±424.9	433.0±304.6	519±302.9	407.0±281.6	a *vs* b = 0.199<br>a *vs* c = 0.228<br>a *vs* d = 0.159
**Leukocytes**	15.7±5.2	12.5±6.9	10.6±6.9	13.6±6.4	a *vs* b = 0.403<br>a *vs* c = 0.195<br>a *vs* d = 0.567
**GOT**	308.3±191.1	281.0±231.9	129.6±56.5	145.7±198.9	a *vs* b = 0.835<br>a *vs* c = 0.072<br>a *vs* d = 0.201
**GPT**	653.7±193.2	486.6±339.9	498.0±228.8	368.0±208.1	a *vs* b = 0.331<br>a *vs* c = 0.252<br>a *vs* d = 0.167
**Creatinine**	0.689±0.5	0.49±0.21	0.67±0.24	0.692±0.567	a *vs* b = 0.421<br>a *vs* c = 0.975<br>a *vs* d = 0.953

Table S2: Biochemical markers of liver function (mean ±SD) was improved after compound **4d** treatment for 24 or 48 hours without any side effect on kidney.

**Parameters**	**CCl_4[a]**	**CCl_4+50 mg/kg/14d[b]**	**CCl_4+ 200 mg/kg/6hrs[c]**	**CCl_4+ 200 mg/kg/24hrs[d]**	**CCl_4+200 mg/kg/48hrs [e]**	***P***
**GOT (AST)**	3667.0±120.1	449.20±109.2	3419.00±130.5	1161.00±108.5	208.40±9.32	a *vs* b = **<0.001**<br>a *vs* c = **0.014**<br>a *vs* d = **<0.001**<br>a *vs* e = **<0.001**
**GPT (ALT)**	3628.0±342.1	2028.6± 168	3430.4±137.48	2209.0±132.8	961.6±15.96	a *vs* b = **<0.001**<br>a *vs* c = 0.282<br>a *vs* d = **<0.001**<br>a *vs* e = **<0.001**
**Creatinine**	0.4240±0.21	0.4120±0.2	0.4560±0.18229	0.4860±0.17	0.4660±0.16	a *vs* b= 0.929<br>a *vs* c = 0.803<br>a *vs* d = 0.624<br>a *vs* e = 0.733

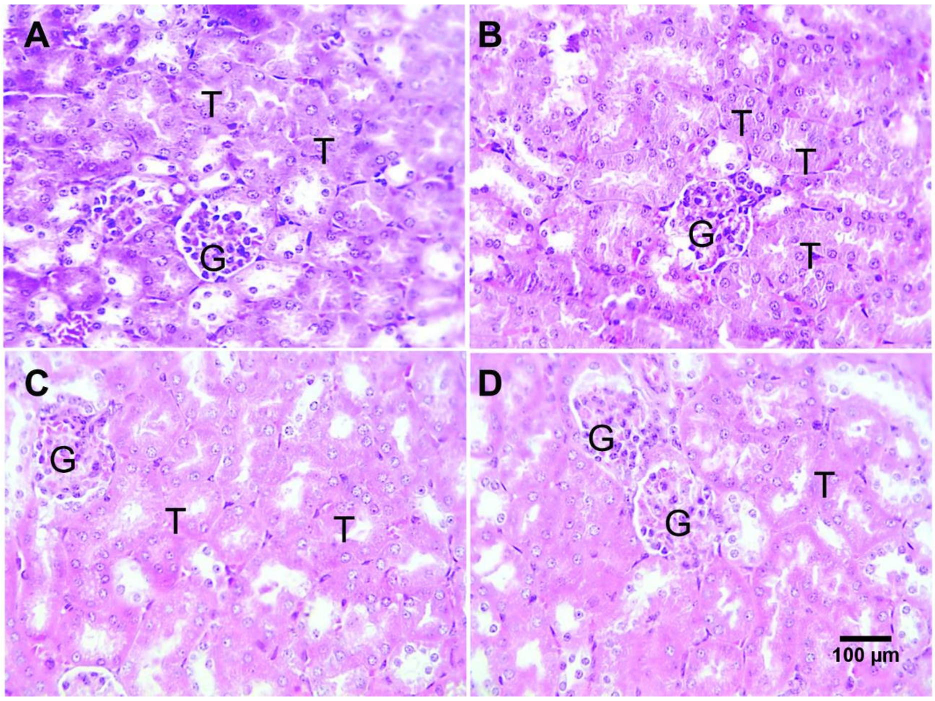

Figure S1: H&E staining of kidney showing no pathological alteration in glomeruli (G) and tubules (T) of mice treated with (A) 10% DMSO, (B) 50 mg/kg compound **4d**, (C) 200 mg/kg/24 hrs compound **4d** and (D) 200 mg/kg/48 hrs compound **4d**. (HE, X: 100)

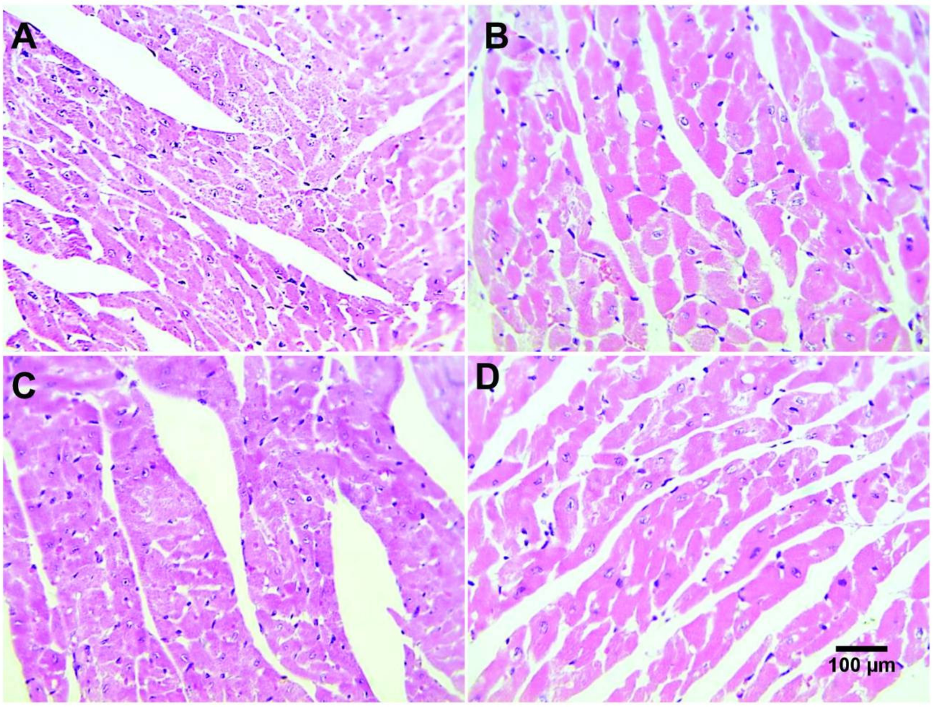

Figure S2: H&E staining of heart showed no pathological alteration in cardiomyocytes of mice treated with (A)10% DMSO, (B) 50 mg/kg compound **4d**, (C) 200 mg/kg/24 hrs compound **4d** and (D) 200 mg/kg/48 hrs compound **4d**. (HE, X: 100)

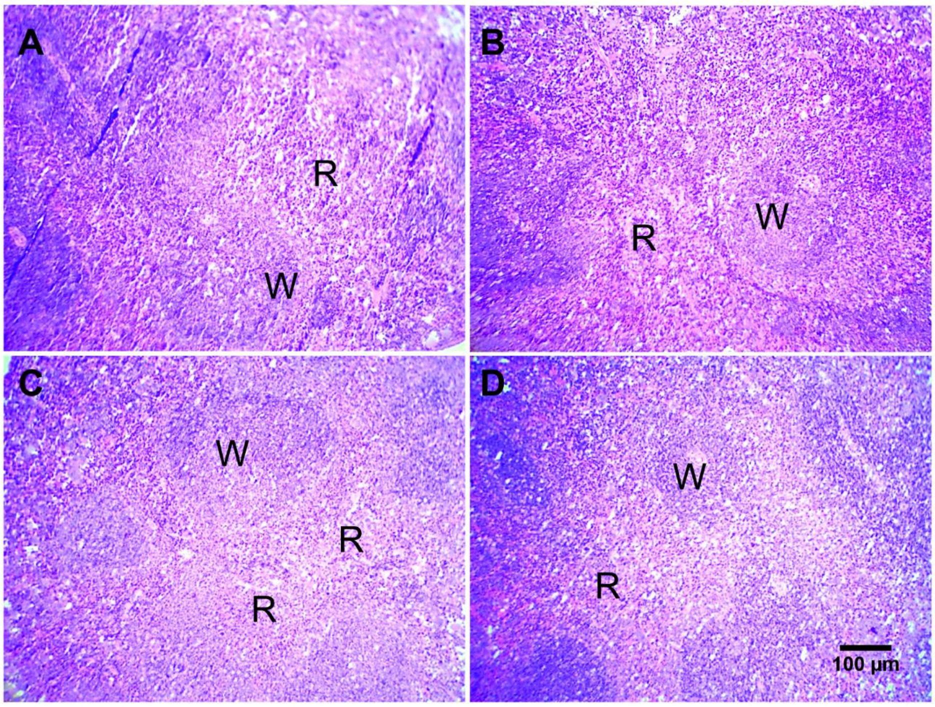

Figure S3: H&E staining of spleen showed no pathological alteration in red pulp (R) and white pulp (W) in mice treated with (A)10% DMSO, (B) 50 mg/kg compound **4d**, (C) 200 mg/kg/24 hrs compound **4d** and (D) 200 mg/kg/48 hrs Compound **4d**. (HE, X: 100).
